# Progressive tooth pattern changes in *Cilk1*-deficient mice depending on Hedgehog signaling

**DOI:** 10.1038/s41368-025-00405-4

**Published:** 2025-12-01

**Authors:** Minjae Kyeong, Ju-Kyung Jeong, Dinuka Adasooriya, Shiqi Kan, Jiwoo Kim, Jieun Song, Sihyeon Park, Suyeon Je, Seok Jun Moon, Young-Bum Park, Hyuk Wan Ko, Eui-Sic Cho, Sung-Won Cho

**Affiliations:** 1 Department of Oral Biology, BK21 FOUR Project, Oral Science Research Center, Yonsei University College of Dentistry, Seoul, South Korea; 2 Cluster for Craniofacial Development and Regeneration Research, Institute of Oral Biosciences, Jeonbuk National University School of Dentistry, Jeonju, South Korea; 3 Department of Biochemistry, Yonsei University College of Life Science and Biotechnology, Seoul, South Korea; 4 Taste Research Center, Yonsei University College of Dentistry, Seoul, South Korea; 5 Department of Prosthodontics, Yonsei University College of Dentistry, Seoul, South Korea

**Keywords:** Developmental biology, Oral diseases

## Abstract

Primary cilia function as critical sensory organelles that mediate multiple signaling pathways, including the Hedgehog (Hh) pathway, which is essential for organ patterning and morphogenesis. Disruptions in Hh signaling have been implicated in supernumerary tooth formation and molar fusion in mutant mice. Cilk1, a highly conserved serine/threonine-protein kinase localized within primary cilia, plays a critical role in ciliary transport. Loss of *Cilk1* results in severe ciliopathy phenotypes, including polydactyly, edema, and cleft palate. However, the role of Cilk1 in tooth development remains unexplored. In this study, we investigated the role of *Cilk1* in tooth development. *Cilk1* was found to be expressed in both the epithelial and mesenchymal compartments of developing molars. *Cilk1* deficiency resulted in altered ciliary dynamics, characterized by reduced frequency and increased length, accompanied by downregulation of Hh target genes, such as *Ptch1* and *Sostdc1*, leading to the formation of diastemal supernumerary teeth. Furthermore, in *Cilk1*^−/−^;PCS1–MRCS1^△/△^ mice, which exhibit a compounded suppression of Hh signaling, we uncovered a novel phenomenon: diastemal supernumerary teeth can be larger than first molars. Based on these findings, we propose a progressive model linking Hh signaling levels to sequential changes in tooth patterning: initially inducing diastemal supernumerary teeth, then enlarging them, and ultimately leading to molar fusion. This study reveals a previously unrecognized role of Cilk1 in controlling tooth morphology via Hh signaling and highlights how Hh signaling levels shape tooth patterning in a gradient-dependent manner.

## Introduction

The primary cilium is a microtubule-based organelle that extends from the surface of most mammalian cells. They act as sensory structures that transduce extracellular signals into intracellular responses, influencing fundamental cellular functions during development and homeostasis.^1,2^ Primary cilia mediate multiple signaling pathways, including Hedgehog (Hh),^3,4^ Wnt,^5^ Fibroblast growth factor,^6^ Hippo,^7^ mTOR,^8,9^ platelet-derived growth factor,^10^ and Notch^11^ pathways. Of these, Hh signaling is most intimately linked to ciliary function and is essential for the patterning of various organs.^3^ Aberrant Hh signaling can lead to supernumerary teeth and molar fusion.^12–14^

Supernumerary teeth in mice are defined as additional teeth that develop beyond the typical complement of one incisor and three molars per quadrant. Based on their location and developmental origin, supernumerary teeth are simply categorized into three types: supernumerary incisors, supernumerary teeth in the toothless diastema region, and supernumerary molars located lingual or distal to the normal molars.^15^ During wild-type molar development, two transient tooth germs, referred to as the anterior diastemal rudiment (R1) and the posterior diastemal rudiment (R2), appear in the diastema region between the incisor and molars. R1 emerges around embryonic day 12.5 (E12.5) and regresses shortly thereafter, while R2 appears at E13.5 and typically regresses by E14.5 through incorporation into the developing first molar (M1), which initiates just distal to R2.^14,16–19^ This integration illustrates that M1 originates from the fusion of multiple tooth primordia, namely the R2 and M1. The second (M2) and third molars (M3) subsequently develop in a posterior sequence. However, in certain mutant mice, the R2 bud fails to regress and instead develops into a supernumerary tooth, which precedes the normal formation of M1 and M2. Thus, the diastemal supernumerary tooth corresponds to R2. Many mutant mouse models with impaired Hedgehog signaling have been reported to develop R2, including *Gas1*^−/−^, *Shh*⁺^/^⁻; *Sostdc1*⁺^/^⁻, *Sostdc1*^−/−^, and mice with deletions of *Shh* enhancers such as MRCS1^△/△^ and PCS1–MRCS1^△/△^ mice.^12,14,20,21^ PCS1–MRCS1^△/△^ mice, lacking PCS1 and MRCS1, two oral-specific cis-regulatory enhancer elements of *Shh* (*Sonic Hedgehog*), exhibit R2 in both the maxilla and mandible.^12^

Molar fusion in mice refers to a developmental anomaly in which two or more adjacent molar tooth germs, most commonly M1 and M2, fail to undergo proper segmentation and instead develop as a single fused molar. Other Hh signaling mutants, such as *Krt14-Cre;Shh*^fl/−^*, Krt14-Cre;Smo*^fl/fl^, and *Sostdc1*^−/−^ mice, also develop molar fusion.^13,14,22–26^

Given the role of primary cilia in Hh signaling, we investigated *Cilk1* (*Ciliogenesis-associated kinase 1*) to understand its contribution to tooth patterning. Cilk1 is a key regulator of ciliary turnaround processes. It localizes at ciliary tips via interactions with the IFT-B complex.^27^ Cilk1 regulates the undocking of kinesin-II from intraflagellar transport (IFT) particles and coordinates the turnaround of the IFT machinery by phosphorylating kinesin-II subunits or other components of the IFT complex.^28–33^ This regulatory function is supported by the abnormal accumulation of IFT components at the ciliary tip in *Cilk1*-deficient cells.^34^ Mutations in *Cilk1* result in elongated cilia and impair ciliary function, leading to defects in Hh signaling and are associated with ciliopathies, a group of syndromes that arise from impairments in sensory and signaling activities of the primary cilia and affect nearly all organs in the body.^34–36^ Cilk1 is critical for the proper retrograde trafficking and ciliary exit of Smoothened (Smo), a ciliary G protein–coupled receptor that mediates Hh signaling. In wild-type cells, stimulation with the Smoothened agonist (SAG) facilitates Smo entry into the primary cilium, resulting in downstream activation of the Hh pathway. By contrast, *Cilk1*-deficient cells exhibit excessive accumulation of Smo within the primary cilium even under unstimulated conditions, without corresponding activation of Hedgehog signaling.^34^ While loss-of-function mutations in primary cilia-related genes such as *Ift88*, *Ofd1*, and *Kif3a* cause severe developmental defects, including cleft palate and abnormal tooth patterning,^37–39^ the specific impact of Cilk1 on tooth development remains unclear.

In this study, we examined *Cilk1* expression in developing tooth germs and analyzed tooth patterning in both conventional and conditional *Cilk1*-deficient mice. Our findings reveal that loss of *Cilk1* results in R2 formation, altered ciliary morphology, and reduced Hh signaling activity. Furthermore, we generated *Cilk1*^*−/−*^; PCS1–MRCS1^△/△^ mice, which exhibited R2 enlargement and molar fusion, reinforcing the link between Hh signaling activity and tooth patterning.

## Results

### *Cilk1* deletion leads to diastemal supernumerary tooth formation

**Fig. 1:**
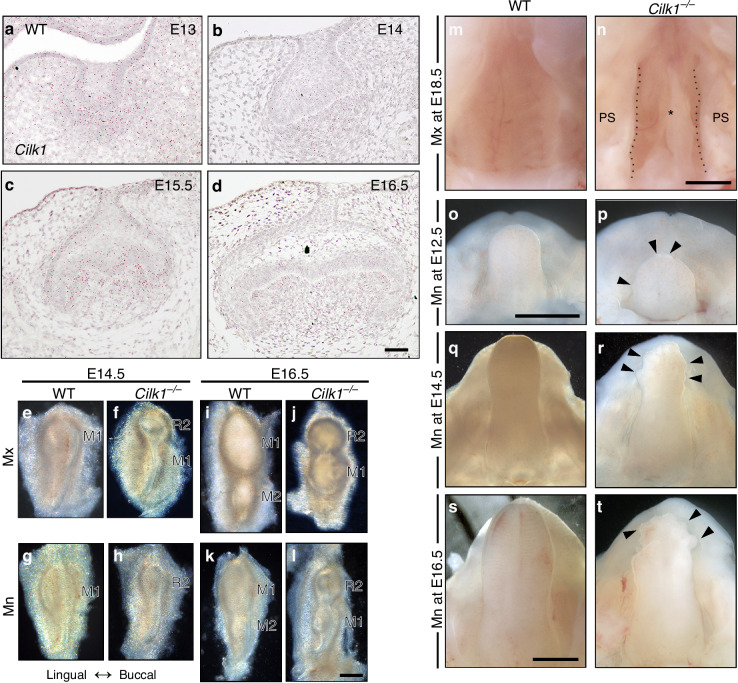
*Cilk1* expression in developing molars and developmental anomalies in *Cilk1*^−/−^ mice. **a**–**d** *Cilk1* expression (small brown dots) in frontal sections of the developing first molar (M1) of wild-type mice at E13, E14, E15.5, and E16.5. *Cilk1* is weakly expressed in dental epithelium and dental mesenchyme. **e**–**l** Tooth germs dissected from maxilla (Mx) and mandible (Mn) of wild-type (WT) and *Cilk1*^*−/−*^ mice at E14.5 and E16.5. Diastemal supernumerary tooth (R2) is observed in *Cilk1*^*−/−*^ mice at E14.5 (**f**, **h**). M1 and second molar (M2) are observed in wild-type mice at E16.5 (**i**, **k**), while R2 and M1 are observed in *Cilk1*^*−/−*^ mice (**j**, **l**). R2 is similar or smaller in size than M1 in both maxilla and mandible of *Cilk1*^*−/−*^ mice at E16.5. **m** Wild-type mice show the fused palate at E18.5. **n** *Cilk1*^−/−^ mice show cleft palate (asterisk) at E18.5 with two palatal shelves (PS). **o**–**t** *Cilk1*^−/−^ embryo shows multiple lobules (black arrowheads) in the tongue at E12.5. Scale bars, **a**–**d**: 50 μm, **e**–**l**: 0.25 mm, **m**–**t**: 1 mm

To investigate the spatiotemporal expression of *Cilk1* during tooth development, we performed RNA in situ hybridization. *Cilk1* expression was detected in both the epithelium and mesenchyme of the mandibular M1 from E13.5 to E16.5 (Fig. 1a–d). To further confirm this pattern, we analyzed recently published single-cell RNA-sequencing (scRNA-seq) data,^40,41^ which demonstrated that *Cilk1* was broadly expressed across both epithelial and mesenchymal compartments at E14.5 and E16.5 (Supplementary Fig. S1b, j). Immunohistochemistry staining also showed the presence of primary cilia in most cells within both dental epithelium and mesenchyme (Fig. 3e, g), confirming that Cilk1 is closely associated with primary cilia during tooth development, as in other organs.

In *Cilk1*^*−/−*^ mice, R2 formation was observed at E14.5 and E16.5. By E14.5, both wild-type and *Cilk1*^*−/−*^ mice had a single molar. However, the *Cilk1*^*−/−*^ molar was significantly smaller (Fig. 1e–h). At E16.5, two molars were visible in wild-type mice, whereas *Cilk1*^*−/−*^ mice exhibited an abnormally smaller mesial molar, indicative of R2 formation (Fig. 1i–l). This aberrant tooth patterning was observed in 100% of *Cilk1*^*−/−*^ mice in both the maxilla and mandible. In frontal sections of wild-type mice, R2 regressed, and M1 consistently reached more advanced developmental stages than M2 at corresponding time points. In contrast, in *Cilk1*^*−/−*^ mice, R2 survived and progressed earlier than M1 (Supplementary Fig. S2a–f”).

Additional craniofacial abnormalities, including cleft palate and lobulated tongue, were detected in *Cilk1*^*−/−*^ mice (Fig. 1m–t), consistent with previous reports.^42^ These defects were observed in 100% of *Cilk1*^*−/−*^ mice, reinforcing the role of Cilk1 in craniofacial and tooth development.

**Fig. 2:**
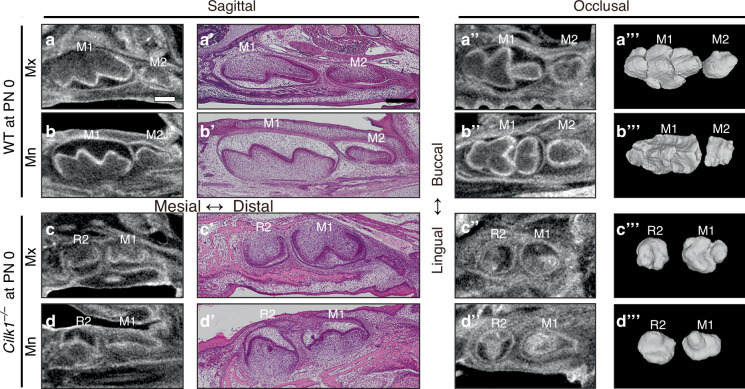
Diastemal supernumerary teeth in *Cilk1*^−/−^ mice. **a**–**d** Micro-computed tomography (micro-CT) sections, histologic images, and 3-dimensional reconstructed tooth germs at PN 0. First molar (M1) is larger than second molar (M2) in wild-type mice in both maxilla (**a**–**a”’**) and mandible (**b**–**b”’**). A smaller-sized diastemal supernumerary tooth (R2) is observed on the mesial side of M1 in *Cilk1*^*−/−*^ mice in both maxilla (**c**–**c”’**) and mandible (**d**–**d”’**). Scale bars, **a**–**d**, **a’**–**d’**: 0.25 mm

**Fig. 3:**
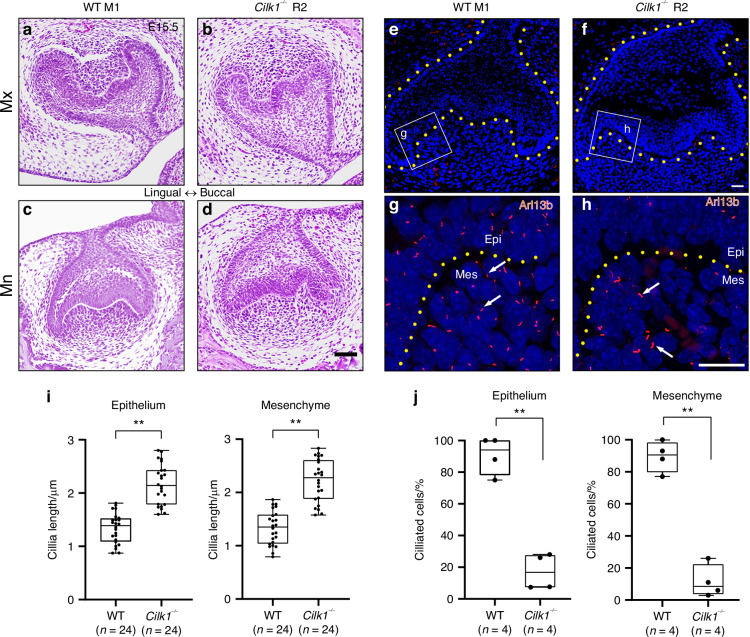
Alterations in primary cilia and gene expression in developing molars in *Cilk1*^−/−^ mice. **a**–**d** Frontal histologic sections of first molar (M1) of wild-type (WT) and diastemal supernumerary tooth (R2) of *Cilk1*^−/−^ mice at E15.5. The dental epithelium of *Cilk1*^−/−^ mice is different in shape from that of wild-type mice. **e**–**h** Immunohistochemistry of Arl13b in tooth germs at the cap stage. Arl13b highlights the cilia (white arrows) in the dental epithelium (Epi) and mesenchyme (Mes) of wild-type and *Cilk1*^−/−^ tooth germs at E15.5. DAPI was used for counterstaining. **i**, **j** Quantification of ciliary length and ciliated cell percentages in the dental epithelium and mesenchyme of wild-type M1 and *Cilk1*^−/−^ R2. Primary cilia are significantly elongated in both the dental epithelium and mesenchyme of R2 (**i**). Percentage of ciliated cells in dental epithelium and mesenchyme is significantly decreased in R2 of *Cilk1*^−/−^ mice (**j**). Scale bars: **a**–**d**: 500 μm, **e**–**h**: 20 μm. **P < 0.01

Due to perinatal lethality, the final tooth pattern was assessed at postnatal day 0 (PN 0) (Fig. 2a–d). At this stage, R2 was retained in both the maxilla and mandible of *Cilk1*^*−/−*^ mice, as confirmed by micro-CT images. Sagittal and occlusal sections revealed that R2 was smaller than M1, indicating an altered developmental trajectory. In frontal sections of molars at E15.5 and PN 0, the dental epithelium of R2 in *Cilk1*^*−/−*^ mice differed from M1 in wild-type mice (Fig. 3a–d, Supplementary Fig. S2g−j). Notably, a thickened dental lamina was observed in mandibular R2 (Supplementary Fig. S2j). Ameloblasts and odontoblasts were well observed in both *Cilk1*^−/−^ and wild-type mice (Supplementary Fig. S2k, l).

### *Cilk1* loss increases primary cilia length and reduces Hh signaling activity

The length of primary cilia and Hh signaling activity were significantly altered in the developing tooth germ of *Cilk1*^*−/−*^ mice. To determine whether the observed changes in tooth pattern were associated with numerical and morphological alterations in primary cilia, we quantified cilia frequency and length in frontal sections of developing molars at E15.5.

Immunohistochemistry staining using Arl13b, a specific ciliary marker, confirmed the presence of primary cilia in the majority of cells within both dental epithelium and mesenchyme (Fig. 3g), consistent with previous findings.^43^ In *Cilk1*^*−/−*^ mice, primary cilia were significantly elongated, whereas their overall frequency was markedly reduced in both epithelial and mesenchymal cells (Fig. 3i, j). These findings indicate that Cilk1 plays a critical role in regulating both ciliary length and frequency during tooth development.

### Mesenchymal *Cilk1* is essential for tooth patterning

**Fig. 4:**
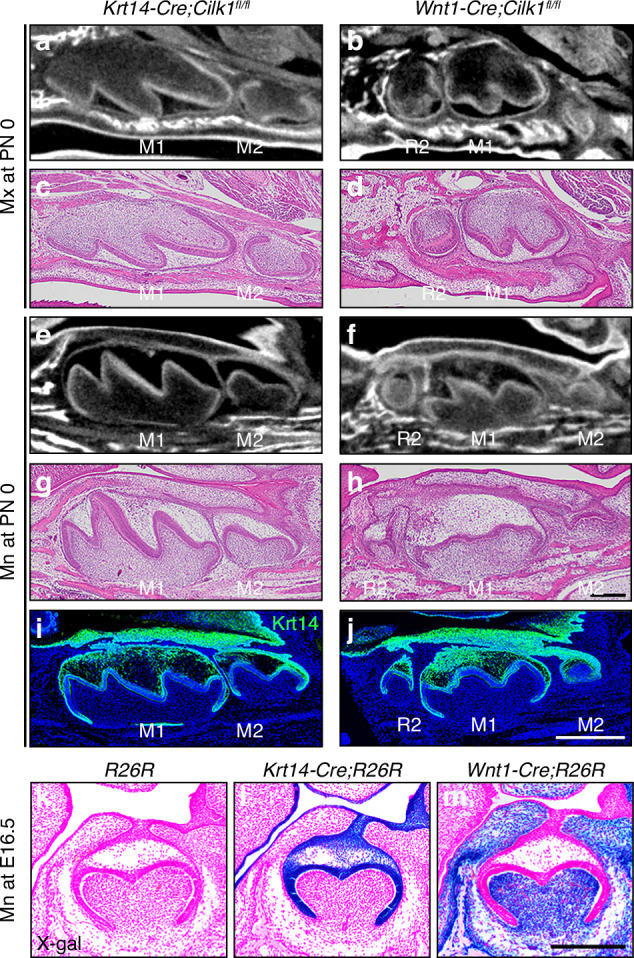
Loss of *Cilk1* in mesenchyme leads to changes in tooth pattern. **a**–**j** Sagittal sections of the maxilla (Mx) and mandible (Mn)of *Krt14-Cre;Cilk1*^fl/fl^ and *Wnt1-Cre;Cilk1*^fl/fl^ mice at PN 0. *Krt14-Cre;Cilk1*^fl/fl^ shows a normal tooth pattern with first molar (M1) and second molar (M2). *Wnt1-Cre;Cilk1*^fl/fl^ shows smaller diastemal supernumerary tooth (R2), M1 and M2. **i**, **j** R2 is clearly observed in *Wnt1-Cre;Cilk1*^fl/fl^ mice by the immunofluorescent staining against Krt14. DAPI was used for counterstaining. At E16.5, X-gal–positive cells were absent in R26R control mice (**k**), present in the oral and dental epithelium of *Krt14-Cre*;R26R mice (**l**) and observed in the oral and dental mesenchyme of *Wnt1-Cre*;R26R mice (**m**) confirming tissue-specific Cre recombinase activity. Scale bars: **a**–**h**: 250 μm, **i**, **j**: 500 μm, **k**–**m**: 200 μm

To determine whether primary cilia in the epithelium or mesenchyme are critical for tooth patterning, we generated tissue-specific *Cilk1*^*−/−*^ mice (Fig. 4).

First, *Krt14-Cre;Cilk1*^fl/fl^ mice, which lack *Cilk1* in the epithelium, exhibited no apparent phenotypic changes in either gross morphology or tooth patterning at PN 0 (Fig. 4a, c, e, g, i). In contrast, *Wnt1-Cre;Cilk1*^fl/fl^ mice, in which *Cilk1* is deleted in cranial neural crest-derived mesenchyme, developed R2 in both the maxilla and mandible (Fig. 4b, d, f, h, j). Interestingly, M2 development was observed in *Wnt1-Cre;Cilk1*^fl/fl^ mice at PN 0; however, M3, which develops from PN 4 in wild-type mice, could not be assessed due to perinatal lethality. Immunostaining with anti-Krt14 antibody further confirmed the presence of R2 in *Wnt1-Cre;Cilk1*^fl/fl^ mice (Fig. 4j).

The tissue-specific gene knockout efficiency of *Krt14-Cre* and *Wnt1-Cre* transgenic mice has been well characterized in previous studies. The *Wnt1-Cre* line has been shown to activate Cre recombinase in approximately 96% of neural crest cell lineages, including both premigratory and migratory populations.^44^ Similarly, the *Krt14-Cre* line has been reported to mediate gene deletion in nearly all keratinocytes of the skin and oral epithelium.^45,46^ To confirm tissue-specific Cre recombinase activity in this study, X-gal staining was performed at E16.5 in *Krt14-Cre*;R26R and *Wnt1-Cre*;R26R embryos (Fig. 4k–m). In *Krt14-Cre*;R26R mice, X-gal–positive cells were detected in the oral and dental epithelium, whereas in *Wnt1-Cre*;R26R mice, X-gal–positive cells were observed in the oral and dental mesenchyme. These patterns confirm epithelial- and mesenchymal-specific Cre activity in the respective transgenic mouse lines. PCR analysis was also performed using epithelial tissues isolated from the tongue and palate of *Krt14-Cre;Cilk1*^fl/fl^ mice at PN 3, confirming that exon 6 of *Cilk1* was efficiently and completely deleted in the oral epithelium by *Krt14-Cre*–mediated recombination (Supplementary Fig. S3a–c). Additionally, while *Krt14-Cre;Cilk1*^fl/fl^ mice were viable beyond birth, *Wnt1-Cre;Cilk1*^fl/fl^ mice exhibited cleft palate, lobulated tongue, and perinatal lethality (Fig. 6d). These findings strongly indicate that mesenchymal primary cilia play a critical role in tooth patterning.

### *Cilk1*^−/−^ tooth germs form calcified teeth

**Fig. 5:**
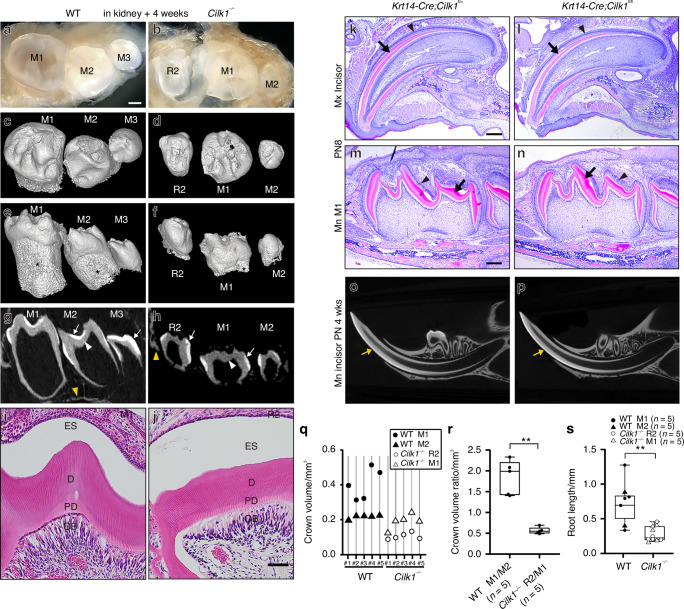
*Cilk1*-deficient tooth germs form fully calcified teeth. **a**, **b** Fully calcified teeth formed from mandibular E14.5 developing molars of wild-type (WT) and *Cilk1*^−/−^ mice within the renal subcapsular layer over a 4-week period. **c**, **d** Micro-CT reconstructions showing distinct differences in tooth pattern between wild-type and *Cilk1*^*−/−*^ mice. In wild-type samples, tooth size progressively decreases from left to right, whereas in *Cilk1*^*−/−*^ calcified teeth, the central tooth is the largest. Wild-type samples show the first, second, and third molars (M1, M2, M3) from left to right, whereas *Cilk1*^*−/−*^ samples show the diastemal supernumerary tooth (R2), M1, and M2. **e**, **f** Comparative images showing that root (asterisks) length appears shorter in *Cilk1*^*−/−*^ calcified teeth than in wild-type teeth. **g**, **h** Micro-CT sections demonstrating well-formed enamel (white arrows), dentin (white arrowheads), and surrounding bone (yellow arrowheads) in both wild-type and *Cilk1*^*−/−*^ teeth. **i**, **j** Histological analysis confirming the presence of enamel space (ES), dentin (D), dentinal tubules, predentin (PD), and odontoblasts (OB) in both genotypes. **k**–**p** Representative images of *Krt14-Cre;Cilk1*^fl/fl^ mice at PN 8 and PN 4 weeks, showing organic enamel (black arrows) and secretory ameloblasts (black arrowheads) at PN 8 and fully mineralized enamel (yellow arrows) at PN 4 weeks, similar to *Krt14-Cre;Cilk1*^fl/+^ mice. **q** Crown volume measurements of calcified teeth indicate that the central tooth in *Cilk1*^*−/−*^ teeth is the largest, supporting the identification of the left tooth as R2. **r** The relative crown volume of R2 to M1 (R2/M1 ratio) in *Cilk1*^*−/−*^ teeth is significantly smaller than the M1 to M2 volume ratio (M1/M2) in wild-type teeth. **s** Root length is shorter in *Cilk1*^*−/−*^ teeth compared to wild-type controls. Scale bars: **a**–**h**: 250 μm, **i**, **j**: 50 μm, **k**, **l**: 300 μm, **m**, **n**: 200 μm. **P < 0.01

The *Cilk1*^*−/−*^ mice exhibit perinatal lethality, preventing the assessment of the final tooth pattern and dental hard tissue formation. To investigate the role of Cilk1 in the later stages of tooth development, mandibular molars at the cap stage were isolated from both wild-type and *Cilk1*^*−/−*^ embryos at E14.5 and transplanted under the renal capsule of adult wild-type mice. After 4 weeks, fully calcified teeth developed from the transplanted tooth germs of both wild-type and *Cilk1*^*−/−*^ mice (Fig. 5a–j).

First, the final tooth pattern differed between wild-type and *Cilk1*^*−/−*^ calcified teeth. In wild-type samples, tooth size progressively decreased from left to right (Fig. 5a, c), whereas in *Cilk1*^*−/−*^ calcified teeth, the central tooth was the largest (Fig. 5b, d). Crown volume measurements further confirmed that, in all *Cilk1*^*−/−*^ samples, the left tooth was consistently smaller than the central tooth (Fig. 5q), supporting the identification of the left tooth as R2. Moreover, the relative crown volume of R2 to M1 (R2/M1 ratio) in *Cilk1*^*−/−*^ teeth was significantly smaller than the M1 to M2 volume ratio (M1/M2) observed in wild-type samples (Fig. 5r). This *Cilk1*^*−/−*^ tooth pattern is consistent with tooth patterns detected in *Cilk1*^*−/−*^ embryos at E16.5 and PN 0 (Figs. 1j, l and 2c, d) and mirrors the tooth pattern with R2 observed in many mutant mice.^12,47,48^ These findings indicate that R2 in *Cilk1*^*−/−*^ embryos was retained and successfully developed into calcified teeth.

Second, dental hard tissue formation was unaffected by *Cilk1* deletion, though root length was shorter in *Cilk1*^*−/−*^ teeth compared to wild-type (Fig. 5f, h, s). Micro-CT analyses revealed well-formed enamel, dentin, and surrounding bone in all teeth derived from both wild-type and *Cilk1*^*−/−*^ tooth germs (Fig. 5g, h). Histological examination further confirmed the presence of dentinal tubules, pre-dentin, and odontoblasts in both wild-type and *Cilk1*^*−/−*^ teeth (Fig. 5i, j). These results suggest that Cilk1 is not required for the formation of enamel and dentin, implying that ameloblasts and odontoblasts can differentiate and function in the absence of *Cilk1*. Consistent with these results, *Krt14-Cre;Cilk1*^fl/fl^ mice exhibited ameloblasts, odontoblasts, enamel, and dentin at PN 8 and mineralized enamel and dentin by PN 4 weeks, mirroring the normal mineralization process observed in wild-type mice (Fig. 5k–p). These findings indicate that despite *Cilk1* deletion in epithelial cells, the differentiation and functional maturation of ameloblasts proceed normally, highlighting the non-essential role of Cilk1 in enamel formation.

### Tooth patterning is dependent on Hh pathway activity

Previous studies have reported that compound mutant mice, such as *Shh*^*+/−*^;MRCS1^△/△^, *Shh*^*+/−*^;MFCS1^△/△^, and *Shh*^*+/−*^;MFCS4^△/△^, generated by crossing *Shh*^*+/−*^ mice with various *Shh* enhancer-deletion mouse lines, exhibit a more pronounced reduction in Hedgehog signaling activity.^21,49^ In this study, to further investigate the impact of Hh signaling activity on tooth patterning, PCS1–MRCS1^△/△^ mice were crossed with *Cilk1*^*−/−*^ mice to generate *Cilk1*^*−/−*^;PCS1–MRCS1^△/△^ mice. In *Cilk1*^*−/−*^ mice, Hh signaling was impaired in Shh-responsive cells, whereas PCS1–MRCS1^△/△^ mice exhibited reduced *Shh* expression within Shh-producing cells due to the deletion of oral-specific Shh enhancers.^12^ In *Cilk1*^*−/−*^;PCS1–MRCS1^△/△^ mice, Hh signaling is likely simultaneously compromised in both Shh-responsive and Shh-producing cells (Supplementary Fig. S5).

**Fig. 6:**
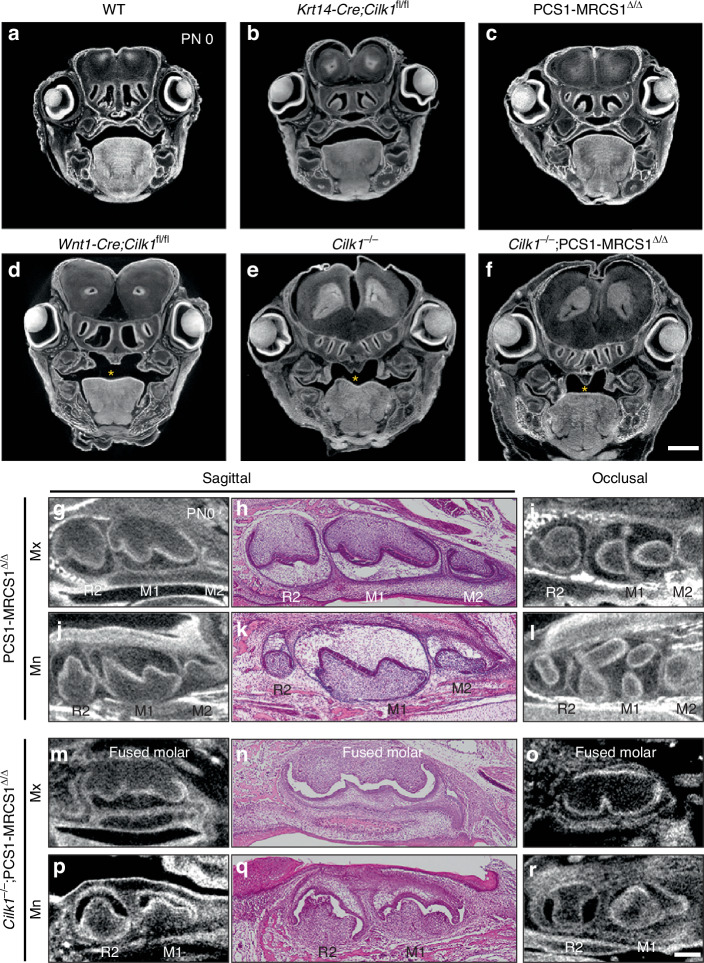
Enlargement of R2 and molar fusion in compound mutant mice. **a**–**f** Frontal sections of the head at eye level in various genotypes at PN 0, showing cleft palate (asterisks) in *Wnt1-Cre;Cilk1*^fl/fl^, *Cilk1*^–/–^ and *Cilk1*^–/–^;PCS1–MRCS1^△/△^ mice. **g**–**r** Micro-CT and histologic images in PCS1–MRCS1^△/△^ and *Cilk1*^*–/–*^;PCS1–MRCS1^△/△^ mice at PN 0. Diastemal supernumerary teeth (R2) are detected in both the maxilla (Mx) and mandible (Mn) of PCS1–MRCS1^△/△^. **m**–**r** *Cilk1*^–/–^;PCS1–MRCS1^△/△^ mice exhibits fused molar in maxilla and R2 and first molar (M1) in mandible

Similar to *Cilk1*^*−/−*^ mice, *Cilk1*^*−/−*^;PCS1–MRCS1^△/△^ mice exhibited perinatal lethality. Additionally, cleft palate and lobulated tongue were observed in *Cilk1*^*−/−*^;PCS1–MRCS1^△/△^ mice, resembling the phenotypes seen in *Wnt1-Cre;Cilk1*^fl/fl^ and *Cilk1*^*−/−*^ mice (Fig. 6a–f).

R2 was also observed in both the maxilla and mandible of *Cilk1*^*−/−*^;PCS1–MRCS1^△/△^ mice at PN 0 (Fig. 6g–r). Moreover, molar fusion was detected in the maxilla but not in the mandible of *Cilk1*^*−/−*^;PCS1–MRCS1^△/△^ mice (Fig. 6m–r).

**Fig. 7:**
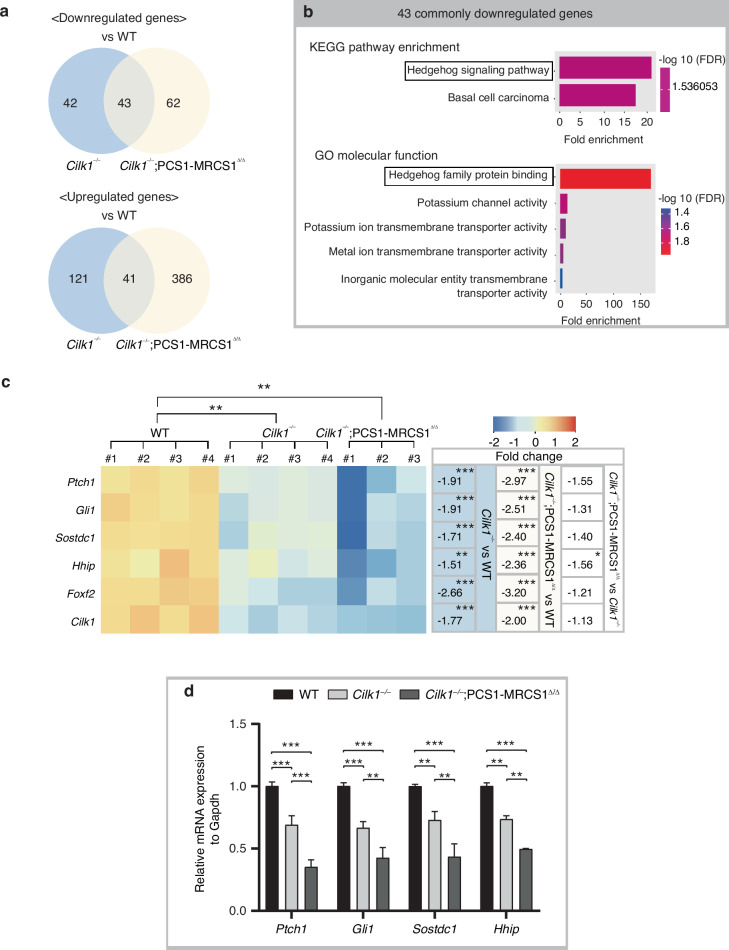
Progressive Hedgehog signaling reduction in mutant mice. **a** Venn diagrams showing the number of differentially expressed genes in *Cilk1*^−/−^ and *Cilk1*^*−/−*^;PCS1–MRCS1^△/△^ mice compared to wild-type (WT) controls at E14.5. Among the significantly downregulated genes, 43 are commonly shared between the two mutant groups. A total of 41 genes are commonly upregulated in both *Cilk1*^−/−^ and *Cilk1*^*−/−*^;PCS1–MRCS1^△/△^ mice compared to WT. **b** Functional enrichment analysis of the 43 commonly downregulated genes showing the significant enrichment of the “Hedgehog signaling pathway” in KEGG and “Hedgehog family protein binding” in Gene Ontology (GO) molecular functions. **c** Heatmaps display the normalized count levels of genes in the *Cilk1*^−/−^, *Cilk1*^*−/−*^;PCS1–MRCS1^△/△^ and wild-type maxillary tooth germs from RNA-sequencing. *Cilk1* and Hedgehog targets genes, such as *Gli1*, *Ptch1, Hhip, Foxf2* and *Sostdc1*, are progressively downregulated from WT to *Cilk1*^−/−^ and further in *Cilk1*^*−/−*^;PCS1–MRCS1^△/△^ mice. Fold changes are calculated relative to wild-type controls. **d** Quantitative PCR analysis showing reduced expression of Hedgehog pathway target genes in *Cilk1*^−/−^ and *Cilk1*^*−/−*^;PCS1–MRCS1^△/△^ mice compared to WT mice at E14.5. **P < 0.01, ***P < 0.001

To further investigate the molecular basis of these alterations, RNA-sequencing was performed on maxillary molars at the cap stage, which are crucial stages for tooth pattern formation. Differential gene expression analysis based on RNA-sequencing revealed 85 significantly downregulated genes in *Cilk1*^*−/−*^ compared to wild type and 105 in *Cilk1*^*−/−*^;PCS1–MRCS1^△/△^ mice. Among these, 43 genes were commonly downregulated in both mutant mice (Fig. 7a, Supplementary Table S2). On the other hand, 41 genes were commonly upregulated in both mutant mice compared to wild-type mice (Fig. 7a, Supplementary Table S1). Functional enrichment analysis of the commonly downregulated genes, compared with wild type, identified significant enrichment of the “Hedgehog signaling pathway” in KEGG pathway analysis and “Hedgehog family protein binding” in Gene Ontology molecular functions (Fig. 7b). *Ptch1*, *Gli1*, *Sostdc1*, *Hhip*, and *Foxf2*, which are known targets of the Hedgehog (Hh) signaling pathway, were among the 43 commonly downregulated genes (Supplementary Fig. S4, Supplementary Table S2). Differential gene expression analysis at E14.5 revealed a progressive reduction in their expression levels from *Cilk1*^*−/−*^ mice to *Cilk1*^*−/−*^;PCS1–MRCS1^△/△^ mice, compared to wild-type controls (Fig. 7c, Supplementary Table S2). This marked reduction in mRNA expression of key Hh target genes was further validated by qPCR (Fig. 7d). These results suggest that Cilk1 is essential for modulating Hh signaling activity during tooth development and that its absence leads to a downregulation of Hh pathway activity, contributing to the observed alterations in tooth patterning. Additionally, RNA-sequencing analysis also confirmed the deletion of *Cilk1* exon 6 in *Cilk1*^*−/−*^ mice and *Cilk1*^*−/−*^;PCS1–MRCS1^△/△^ mice (Supplementary Fig. S6).

**Fig. 8:**
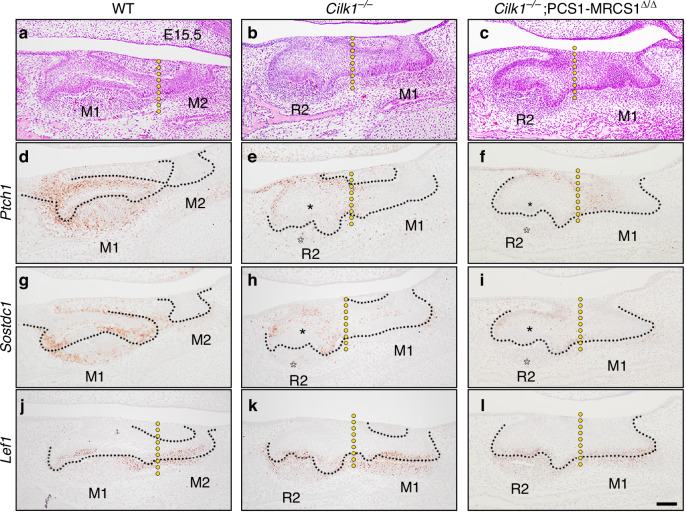
Altered spatial gene expression patterns in tooth germs of *Cilk1* mutant mice. **a**–**c** The sagittal histologic sections of wild-type, *Cilk1*^−/−^, and *Cilk1*^*−/−*^;PCS1–MRCS1^△/△^ mice. R2 in *Cilk1*^−/−^, and *Cilk1*^*−/−*^;PCS1–MRCS1^△/△^ is smaller than first molar (M1) in wild-type mice at E15.5. **d**–**l** *Ptch1* and *Sostdc1* expression were observed in the dental epithelium and mesenchyme of the first molar in wild-type mice, but weakened and absent in the dental epithelium (white asterisks) and mesenchyme (black asterisks), respectively, of R2 in *Cilk1*^−/−^ and *Cilk1*^*−/−*^;PCS1–MRCS1^△/△^ mice. *Lef1* is expressed in all tooth germs and shows non-significant changes between wild-type, *Cilk1*^−/−^, and *Cilk1*^*−/−*^;PCS1–MRCS1^△/△^ mice. Black dotted lines indicate the boundary between epithelium and mesenchyme. Yellow dotted lines indicate the region between M1 and M2

To examine the expression patterns of genes involved in the Wnt-Shh-Sostdc1 negative feedback loop, which is known to regulate tooth patterning,^13^ we performed RNA in situ hybridization to detect expression changes in *Lef1, Ptch1*, and *Sostdc1* in sagittal sections of *Cilk1*^*−/−*^ and *Cilk1*^*−/−*^;PCS1–MRCS1^△/△^ mice at E15.5 (Fig. 8a–l). *Ptch1* and *Sostdc1*, key Hh pathway targets, were weakly expressed in the dental epithelium and completely absent in the dental mesenchyme of R2 in *Cilk1*^*−/−*^ and *Cilk1*^*−/−*^;PCS1–MRCS1^△/△^ mice, compared to wild-type mice (Fig. 8d–i). Conversely, *Lef1* expression remained similar among wild-type, *Cilk1*^*−/−*^ and *Cilk1*^*−/−*^;PCS1–MRCS1^△/△^ tooth germs (Fig. 8j–l). Collectively, these findings suggest that the downregulation of Hh signaling activity is a key driver of R2 formation in *Cilk1*^*−/−*^ and *Cilk1*^*−/−*^;PCS1–MRCS1^△/△^ mice.

**Fig. 9:**
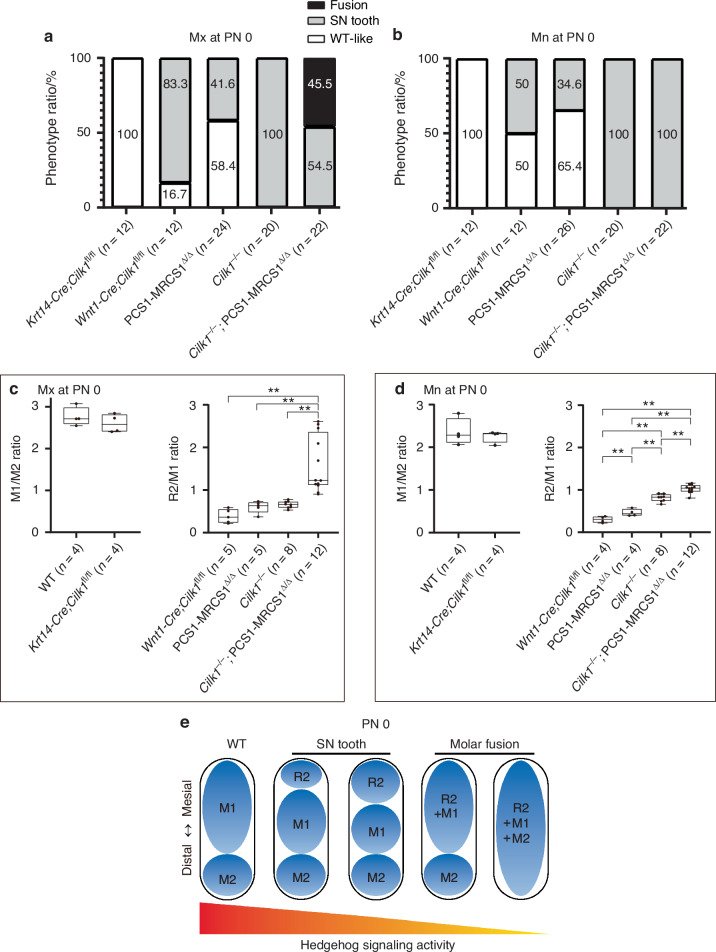
Tooth pattern changes along a gradient of Hedgehog signaling activity. **a**, **b** Quantification of each phenotype penetrance in various genotypes. *Cilk1*^–/–^ mice display 100% penetrance of R2 in both maxilla and mandible. In *Cilk1*^–/–^;PCS1–MRCS1^△/△^ mice, R2 formation and molar fusion are observed in 54.5% and 45.5% of maxilla, respectively, while the mandible exclusively exhibits R2 formation in all cases. While *Krt14-Cre;Cilk1*^fl/fl^ exhibited a WT-like phenotype with no changes in tooth patterning, *Wnt1-Cre;Cilk1*^fl/fl^, PCS1–MRCS1^△/△^, *Cilk1*^–/–^, and *Cilk1*^–/–^;PCS1–MRCS1^△/△^ all displayed patterns of R2 formation. However, a molar fusion phenotype was observed only in the maxilla of *Cilk1*^–/–^;PCS1–MRCS1^△/△^. **c**, **d** Graph showing the relative size of M1 to second molar (M1/M2 ratio) in wild-type and *Krt14-Cre;Cilk1*^fl/fl^ mice and the relative size of R2 to M1 (R2/M1 ratio) in *Wnt1-Cre;Cilk1*^fl/fl^, *Cilk1*^–/–^, and *Cilk1*^–/–^;PCS1–MRCS1^△/△^ mice at PN 0. The size ratio of R2 relative to M1 increases significantly and progressively from *Wnt1-Cre;Cilk1*^fl/fl^ mice to *Cilk1*^*−/−*^ mice, and further to *Cilk1*^*−/−*^;PCS1–MRCS1^△/△^ mice. The mandibular R2/M1 ratio in *Cilk1*^–/–^;PCS1–MRCS1^△/△^ mice ranges from 0.90 to 1.16, whereas the maxillary R2/M1 ratio exhibits broader variability, ranging from 0.90 to 2.61. **e** A hypothetical model for tooth pattern formation influenced by Hh signaling: exploring how R2 and M1 interact. **P < 0.01

Quantitative analysis of R2 formation revealed a 41.6% penetrance in the maxillary quadrants and 34.6% in the mandibular quadrants of PCS1–MRCS1^△/△^ mice. In contrast, *Wnt1-Cre;Cilk1*^fl/fl^ mice exhibited an 83.3% penetrance in the maxillary quadrants and 50% in the mandibular quadrants, while *Cilk1*^*−/−*^ mice consistently showed 100% penetrance in both the maxilla and mandibular quadrants. In *Cilk1*^*−/−*^;PCS1–MRCS1^△/△^ mice, R2 formation and molar fusion were observed in 54.5% and 45.5% of maxillary quadrants, respectively, while the mandibular phenotype exclusively consisted of R2 formation (100% penetrance) (Fig. 9a, b). These findings indicate that as Hh pathway activity decreases, the frequency of R2 formation and molar fusion increases.

Furthermore, as Hh pathway activity decreased, the relative size of R2 to M1 (R2/M1 ratio) progressively increased. The R2/M1 ratio significantly increased across *Wnt1-Cre;Cilk1*^fl/fl^ mice, *Cilk1*^*−/−*^ mice, and *Cilk1*^*−/−*^;PCS1–MRCS1^△/△^ mice. In the mandible of *Cilk1*^*−/−*^;PCS1–MRCS1^△/△^ mice, the R2/M1 ratio ranged from 0.90 to 1.16 (Fig. 9d), and the R2/M1 ratio in the maxilla of *Cilk1*^*−/−*^;PCS1–MRCS1^△/△^ mice varied widely between 0.90 and 2.61 (Fig. 9c), suggesting that as Hh signaling activity decreases, R2 becomes increasingly larger relative to M1. Notably, R2 exceeded the size of M1 at low levels of Hh activity, indicating a gradient-dependent relationship between Hh pathway suppression and tooth patterning abnormalities.

## Discussion

### Cilk1 and craniofacial development

The significance of primary cilia in craniofacial development is well established, as mutations in primary cilia-related genes in both humans and mice frequently result in cleft lip, cleft palate, oligodontia, or supernumerary teeth. In humans, mutations in *EVC*, *EVC2*, *IFT88*, *KIF3A*, *OFD1*, and *IFT121* have been implicated in these craniofacial defects.^50–55^ Similarly, in mice, deletions of *Evc*, *Evc2*, *Fuz*, *Ift88*, or *Kif3a* frequently cause a combination of cleft palate, tongue agenesis, mandibular hypoplasia, and R2 formation.^47,48,56–60^ While cleft palate and lobulated tongue had previously been reported in *Cilk1*-deficient mice,^42^ the present study provides the first evidence of R2 formation in this mutant. Additionally, R2 was observed in *Wnt1-Cre;Ift88*^fl/fl^ and *Wnt1-Cre;Kif3a*^fl/fl^ mice, whereas *Krt5-Cre;Ift88*^fl/fl^ mice exhibited no changes in tooth patterning.^48,61^ These findings, together with our results of R2 formation in *Wnt1-Cre;Cilk1*^fl/fl^ but not in *Krt14-Cre;Cilk1*^fl/fl^ mice, further indicate that mesenchymal primary cilia play a critical role in tooth patterning.

### Cilk1 and the role of primary cilia in tooth development

Molar tooth germ of *Cilk1*^*−/−*^ mice, when transplanted into the subcapsular layer of the kidney, formed the calcified teeth and surrounding bone at 4 weeks after transplantation. These *Cilk1*-deficient teeth showed significantly shorter root length compared to wild-type teeth. This is consistent with previous results that *Ift80*, or *Ift140*-deficient mice, which also have defects in primary cilia, show significantly short roots,^62,63^ suggesting an important role of primary cilia in tooth root growth.

The importance of primary cilia in dental hard tissue formation has been previously reported. Tooth germs from *Wnt1-Cre;Kif3a*^fl/fl^ mice, when transplanted into the kidney capsule, demonstrated the formation of odontoblasts, but with significantly thinner dentin and no enamel,^64^ and *Ift80*, *Ift88*, or *Ift140*-deficient mice showed thin dentin or enamel formation.^61–63^ Our findings in the present study contrast with observations from previous studies. Enamel, dentinal tubules, pre-dentin, dentin, surrounding bone, and odontoblasts in dental pulp were observed in these *Cilk1*^*−/−*^ teeth. Additionally, *Krt14-Cre;Cilk1*^fl/fl^ mice exhibited calcified teeth without any phenotypic changes. These findings suggest that *Cilk1* might not be essential for ameloblasts and odontoblasts differentiation and their hard tissue formation. However, analyzing the shape of the crown and root, as well as the volume of hard tissue, in teeth developed through culture in the kidney is challenging due to spatial constraints within the renal capsule. Therefore, the precise role of *Cilk1* in hard tissue formation should be further investigated.

### Cilk1, Hh signaling, and tooth patterning

Mutations in primary cilia-related genes often result in similar phenotypic alterations due to their central role in Hh signaling.^3,4,60,64,65^ Prior studies have established a strong correlation between Cilk1 and Hh signaling in various organs, including the central nervous system, smooth muscle patterning, and palatal fusion.^28,66,67^ The present study extends this correlation to tooth development by demonstrating a significant reduction in Hh signaling activity in *Cilk1*-deficient tooth germs.

Different tooth patterns arise depending on Hh signaling activity. For example, PCS1–MRCS1^△/△^ mice and MRCS1^△/△^;MFCS4^△/△^ mice, in which *Shh* enhancers were removed, develop R2.^12,21^ In contrast, *Krt14-Cre;Shh*^fl/−^ and *Krt14-Cre;Smo*^fl/fl^ mice exhibit molar fusion.^22,23^ Furthermore, inhibition of Hh signaling through anti-Shh antibodies or Hh pathway inhibitors has been shown to induce supernumerary tooth formation and molar fusion.^13,26^

Phenotypic alterations in tooth pattern can emerge or become more pronounced in compound mutant mice carrying mutations in two different genetic backgrounds. For instance, *Sostdc1*^*+/−*^*;Shh*^*+/−*^ mice, generated by crossing *Sostdc1*^*+/−*^ and *Shh*^*+/−*^ mice with normal tooth patterns, exhibited R2.^14^ Additionally, *Wnt10a*^*+/−*^ and *Wnt10b*^*−/−*^ mice exhibit a normal tooth pattern, and *Wnt10a*^*+/−*^*;Wnt10b*^*−/−*^ mice develop R2.^68^ However, it has remained unclear whether molar fusion can be induced in compound mutant mice combining different mutational backgrounds, all of which have R2. In this study, we demonstrated that *Cilk1*^*−/−*^;PCS1–MRCS1^△/△^ mice developed fused molars in the maxilla. This synergistic effect is due to the simultaneous suppression of Hh signaling in both Shh-producing and Shh-responsive cells (Supplementary Fig. S6). The progressive suppression of Hh signaling from wild-type to *Cilk1*^*−/−*^ mice and further to *Cilk1*^*−/−*^;PCS1–MRCS1^△/△^ mice was validated by differential gene expression analyses, including RNA-sequencing and quantitative PCR. These findings strongly suggest that R2 formation and molar fusion occur progressively as Hh signaling activity declines.

Our study revealed distinct differences in susceptibility to R2 formation and molar fusion between the maxilla and mandible. R2 formation was more prevalent in the maxilla than in the mandible of *Wnt1-Cre;Cilk1*^fl/fl^ and PCS1–MRCS1^△/△^ mice. In contrast, a previous study reported a higher prevalence of R2 in the mandibular quadrants (92%) compared to the maxillary quadrants (58%) in MRCS1^△/△^;*Shh*^+/−^ mice, which lack a distinct *Shh* enhancer.^21^ A similar discrepancy has been documented in *Spry2* and *Spry4* mutant mice, which are negative regulators of the FGF signaling pathway. Among 55 *Spry2*^−/−^ mice analyzed, 97% exhibited R2 formation in the mandible, whereas fewer than 5% showed R2 in the maxilla. In contrast, *Spry4*^−/−^ mice showed R2 formation in 17% of maxillary and only 3% of mandibular quadrants.^69,70^ These findings underscore the need for further systematic investigations into the frequency and distribution of R2 formation across jaw regions. Additionally, in *Cilk1*^*−/−*^;PCS1–MRCS1^△/△^ mice, molar fusion was exclusively observed in the maxilla, consistent with findings in *Evc* and *Evc2*-deficient mice. *Evc-*deficient mice exhibit molar fusion with a penetrance of 100% in the maxilla but only 17% in the mandible.^58^ Similarly, *Evc2* deficiency results in molar fusion restricted to the maxilla, while only R2 formation develops in the mandible.^60^ Comparable jaw-specific differences have also been reported in *Sostdc1*^−/−^ mice. *Sostdc1* encodes a Wnt signaling inhibitor and is a downstream target of the Hedgehog pathway. In *Sostdc1*^−/−^ mice, molar fusion occurs exclusively in the maxilla without accompanying R2, whereas in the mandible, R2 formation is significantly more frequent than molar fusion.^71,72^ The mechanisms underlying these regional differences are unknown but may be related to gene expression and signaling differences between the maxilla and mandible. One plausible explanation for the consistent maxilla-specific occurrence of molar fusion involves differential expression of Wnt inhibitors. Previous study has shown that the Wnt antagonists *Dkk2* and *Wif1* are expressed at significantly higher levels in the mandibular molar mesenchyme compared to the maxillary counterpart in wild-type embryos.^73^ It was proposed that these elevated levels may contribute to the more pronounced mandibular dental defects observed in *Wnt1-Cre;Bmp4*^fl/fl^ mice. By extension, in *Cilk1*^*−/−*^;PCS1–MRCS1^△/△^ and *Sostdc1*^*−/−*^ mice, the mandibular enrichment of alternative Wnt inhibitors such as *Dkk2* and *Wif1* may partially compensate for the loss of *Sostdc1*, thereby reducing the likelihood of molar fusion in the mandible.

### Progressive enlargement of diastemal supernumerary tooth under Hh signaling suppression

Diverse tooth patterns have been reported in many mutant mice, but understanding the mechanism of tooth patterning with significantly reduced Hh or Wnt signaling activity remains challenging. For example, both *Gas1*^*+/–*^ mice and *Shh*^*GFP/+*^ mice develop a normal tooth pattern, while approximately 80% of *Gas1*^*–/–*^ mice exhibit R2. Surprisingly, the penetrance of R2 formation decreases to 38% in *Gas1*^*–/–*^;*Shh*^*GFP/+*^ mice.^20^ Similarly, both *Wnt10a*^*+/−*^ and *Wnt10b*^*−/−*^ mice have a normal tooth pattern, whereas 80% of *Wnt10a*^*+/−*^;*Wnt10b*^*−/−*^ mice develop R2. Remarkably, once more, *Wnt10a*^−/−^;*Wnt10b*^−/−^ mice, despite displaying significantly smaller molars, maxilla, mandible, and body length, exhibit a normal-like tooth pattern. Since *Wnt10a*^−/−^;*Wnt10b*^−/−^ mice retain three molars per jaw quadrant, with the most mesial molar notably larger than the next molar, their tooth pattern was classified as lacking R2.^68^

Comparable patterns were observed in this study. *Wnt1-Cre;Cilk1*^fl/fl^, PCS1–MRCS1^△/△^, and *Cilk1*^*−/−*^ mice exhibited R2, which were consistently smaller than M1 within the same quadrant. However, in *Cilk1*^*−/−*^;PCS1–MRCS1^△/△^ mice, the R2 was equal to or larger than M1. The size ratio of R2 relative to M1 increased significantly and progressively from *Wnt1-Cre;Cilk1*^fl/fl^ mice to *Cilk1*^*−/−*^ mice, and further to *Cilk1*^*−/−*^;PCS1–MRCS1^△/△^ mice.

These findings suggest that a reduction in Hh signaling activity promotes R2 formation and progressively increases the relative size of R2. Under conditions of markedly diminished Hh signaling, R2 may exceed the size of M1. Reanalyzing results from *Gas1*^–/–^;*Shh*^GFP/+^ mice and *Wnt10a*^*−/−*^;*Wnt10b*^*−/−*^ mice, the most mesial molar in each quadrant is likely an R2 rather than M1. Moreover, the normal-like tooth pattern observed in these mice should not be interpreted as a rescued phenotype, but rather as an exacerbated phenotype.

### A progressive model from diastemal supernumerary tooth to molar fusion

In this study, we identified a unique tooth pattern characterized by R2 enlargement. In some *Cilk1*^*−/−*^;PCS1–MRCS1^△/△^ mice, the R2 in the maxilla was approximately 2.5 times larger than M1 and comparable in size to the fused molar. These findings suggest that extreme enlargement of R2 may serve as a precursor to molar fusion. We propose a progressive model in which the R2 retains, enlarges, and subsequently fuses with M1 as Hh signaling activity declines.

This progressive model can be analyzed by the Wnt-Shh-Sostdc1 negative feedback loop, which is the previously suggested mechanism for tooth pattern formation.^12–14^ In wild-type mice, the R2 present at E13 degenerates due to inhibitors (Sostdc1) secreted by M1, resulting in the three-molar pattern. However, in mice with diminished Hh signaling, the R2 retains rather than regressing, as the reduced activity of mediators (Shh signaling) in M1 leads to lower inhibitor levels. As Shh signaling activity continues to decline, the mutual inhibitory effects between developing R2 and M1 weaken, allowing the R2 to expand. With further Hh pathway suppression, inhibitors are lost in all molars, leading to a blurred boundary between adjacent molars and eventual molar fusion (Fig. 9e). A broader spectrum of tooth pattern diversity in other signaling pathways, in addition to the Hh pathway, will provide further support for the progressive model.

Collectively, our findings highlight the critical role of Cilk1 in primary cilia function and the importance of Hh signaling during tooth development. We uncover a new insight into tooth patterning dynamics: R2 can be larger than M1. Furthermore, we propose a progressive model where R2 formation, R2 enlargement, and ultimately tooth fusion occur sequentially in response to the degree of Hh pathway suppression.

## Materials and methods

### Animals

All animal experiments were performed under approved protocols of Yonsei University Health System Institutional Animal Care and Use Committee (YUHS-IACUC). All mice used in this study were euthanized via CO_2_ exposure at selected embryonic and postnatal days. A mix of males and females was assigned without considering the sex, and at least one individual from each sex was included in each group after screening for the genotype with PCR. All mice were maintained in the C57BL/6 background. PCS1–MRCS1^△/△^ mice with a deletion of a 70 kb region from PCS1 to MRCS1 were previously described.^12^ *Cilk1*^*−/−*^, *Cilk1*^fl/fl^, *Wnt1-Cre*, and *Krt14-Cre* mice were described previously.^22,28,42,66,74,75^ In brief, *Cilk1*^fl/fl^ mice were generated by crossing *Cilk1*^*tm1a(KOMP)Mbp*^ mice with Rosa26-FLP1 mice, which express FLP1 recombinase under the control of Rosa26. To generate *Cilk1*^−/−^ mice, *Cilk1*^fl/fl^ mice were crossed with *EIIa-Cre* transgenic mice, enabling Cre-mediated deletion of *Cilk1* exon 6 in germ cells. *Cilk1*^–/–^ mice were subsequently crossed with PCS1–MRCS1^△/△^ mice to generate *Cilk1*^–/–^;PCS1–MRCS1^△/△^ mice. For tissue-specific deletion, *Cilk1*^fl/fl^ mice were crossed with *Krt14-Cre* and *Wnt1-Cre* transgenic mice to obtain epithelial- and mesenchyme-specific *Cilk1* knockout mice, respectively (Supplementary Fig. S8).

### Section in situ hybridization

Heads were dissected from wild-type mouse embryos at E13, E14, E15.5, and E16.5 on cold DEPC-PBS and fixed with 4% PFA in DEPC-PBS overnight at 4 °C with rocking. After being rinsed in DEPC-PBS, the samples were decalcified for 3 h at 4 °C using 10% EDTA in DEPC-PBS. Decalcified samples were washed with DEPC-PBS, followed by a saline wash. Samples were dehydrated with a graded ethanol series. Embryo samples were embedded in paraffin and sectioned at 5 µm thickness. Advanced Cell Diagnostics (Newark, USA) designed and manufactured antisense Cilk1 RNA probes. The RNAscope® 2.5 High Definition (HD) assay-brown (Advanced Cell Diagnostics) was used for in situ hybridization in accordance with the manufacturer’s instructions for 322452 (FFPE sample preparation and pretreatment) and 322310 (RNAscope® 2.5 HD Detection Reagent-brown). Mm-Cilk1 (1177181, targeting NM_001163780.1, nucleotide 1358-2353), Mm-Ptch1 (402811, targeting NM_008957.2, nucleotide 2260-3220), Mm-Sostdc1 (313151, targeting NM_025312.3, nucleotide 22-1385), Mm-Lef1 (441861, targeting NM_175263.4, nucleotide 406-1623) RNA probes were used.

### Histological and immunohistochemistry staining

Heads were fixed with 4% PFA at 4 °C for 24 h and decalcified in 10% ethylenediaminetetraacetic acid (EDTA) at 4 °C for 24 h. The tissues were then dehydrated through a graded ethanol series, embedded in paraffin, and sectioned at 5 or 7 µm thickness. For histological analysis, sections were stained with hematoxylin and eosin using standard protocols. For immunohistochemistry staining, sections underwent antigen retrieval by incubation in citrate buffer (pH 6.0) at 90 °C for 1 h, followed by blocking with 5% bovine serum albumin (BSA) in phosphate-buffered saline with Tween 20 (PBST) at room temperature for 40 min. Sections for primary cilia staining were incubated overnight at 4 °C with Arl13b antibody.^76^ Sections for epithelial staining were incubated overnight at 4 °C with a primary anti-cytokeratin 14 antibody (1:200, LS-B8270, LSBio), washed with PBST, and then incubated for 1 h at room temperature with an Alexa Fluor-conjugated secondary antibody (1:200, Invitrogen). Following antibody incubation, sections were washed and counterstained with DAPI (6-diamidino-2-phenylindole, Sigma) to visualize the nuclei. Images were captured using a fluorescence microscope (Leica Microsystem) or a Zeiss LSM 900 confocal microscope. The ciliary length (*n* = 24 per group) was measured using the Zeiss Zen 3.3 (blue edition) program in a single plane (7 µm thickness) of confocal microscopy Z-stack images. The percentage of ciliated cells was determined by counting DAPI-positive nuclei and Arl13b-positive primary cilia in sections (*n* = 4 per group).

### Single-cell RNA-sequencing analysis

The scRNA-seq datasets for E14.5 and E16.5 mouse mandibular molars were processed, analyzed, and visualized using publicly available Gene Expression Omnibus data (GSE189381 and GSE162413).^40,41^ We imported the pre-processed count matrices into the Biomage (https://biomage.net/)-hosted instance of Trailmaker^®^ (https://app.trailmaker.parsebiosciences.com/). Data handling and cell clustering were described previously.^77^ All cell clusters were manually annotated based on existing literatures.^40,41^ The differentially expressed genes in each cluster were listed in the cluster map and heatmap previously.^77^

### Three-dimensional (3D) reconstruction using computed tomography

The dissected embryo heads were stained with phosphotungstic acid (P4006-10G, Sigma-Aldrich) and scanned by a micro-computed tomography (CT) system (Skyscan 1173; Bruker, Kontich, Belgium) using 90 kV, 88 μA, 360° rotation with a step of 0.3°, pixel size of 6 μm and an aluminum filter (1.0 mm of thickness) and reconstructed by the NRecon software (Bruker). The reconstructions were visualized and converted to 3D volumes using the software 3D Slicer.^78^ Crown volume (*n* = 5 per group) and root length (*n* = 5 per group) were measured directly from the 3D reconstructed images.

### RNA-sequencing analysis

Maxillary molars were dissected from mouse embryos at E14.5 (*n* = 6 per biological replicate, and *n* = 4 for wild-type and *Cilk1*^–/–^ group, and *n* = 3 for *Cilk1*^–/–^;PCS1–MRCS1^△/△^ group) in DEPC-PBS. The 0.5 mm zirconia beads were used to homogenize tooth germ samples in TRIZOL^®^ (Invitrogen) using the Bullet Blender® homogenizer (Next Advance, USA). Chloroform was used to phase separate the total RNA, and isopropyl alcohol was used to precipitate it. After being cleaned with 75% ethanol, the RNA pellet was eluted using RNase-free water. NanoDrop (Thermo Fisher Scientific, USA) was used to measure the concentration and quality of RNA. After the library was built and sequenced on an Illumina platform, paired-end reads were produced, quality-controlled, and mapped to the reference genome (mm10). The number of reads mapped to each gene was quantified using FeatureCounts v1.5.0-p3, and FPKM values were calculated based on gene length and read counts. Differential expression analysis between the two conditions was performed using the edgeR package (3.22.5), with read counts adjusted by a scaling normalization factor. *P* values were adjusted using the Benjamini–Hochberg method, and a corrected Padj value of 0.05 and an absolute fold change of 1.5 were set as the threshold for significance. Gene Ontology (GO) enrichment analysis of differentially expressed genes was carried out using ShinyGo 0.77 (http://bioinformatics.sdstate.edu/go/). GO terms with corrected *P* values below 0.05 were considered significantly enriched.

### Statistical analysis

The percentage of ciliated cells was analyzed using the Mann–Whitney *U* test, while the ciliary length and M1/M2 ratio were analyzed using Student’s *t*-tests (two-tailed). The R2/M1 ratio was assessed using one-way analysis of variance (ANOVA) with Tukey’s test for multiple comparisons. Every graph was visualized with Prism 8.0 (GraphPad, San Diego, CA, USA). All data are presented as mean ± SEM. *P* value less than 0.05 was considered a statistically significant difference. *P* value larger than 0.05 was considered a statistically non-significant difference.

### Transplantation of tooth germs into the renal subcapsular layer

Developing molars (*n* = 5 per group) were isolated from mandibles of mouse embryos at E14.5, transplanted into the renal subcapsular layer of C57BL/6 adult male mice, and harvested after 4 weeks. All surgical procedures were performed under anesthesia administered intraperitoneally. No immunosuppressive medication was used.

### Isolation of oral epithelium from tongues and palates in *Krt14-Cre;Cilk1*^fl/fl^ mice

Mucosa from the tongue and palate of *Krt14-Cre;Cilk1*^fl/fl^ mice at PN 3 were dissected and incubated in Dispase II (1.2 U/mL in PBS) for 30 min. After incubation, the tissues were washed in DMEM supplemented with 10% FBS, and the oral epithelium was carefully separated from the underlying mesenchyme. gDNA was extracted from these epithelia, and genotyping PCR was carried out across three individuals.

### RNA extraction and quantitative RT–PCR

Total RNA was extracted from E14.5 embryonic tooth germs (*n* = 6 per biological replicate and triplicate per group) using TRIzol reagent (Qiagen). One microgram of RNA was converted to complementary DNA (cDNA) using the First-Strand cDNA Synthesis Kit (Takara) according to the manufacturer’s instructions. Quantitative reverse transcription PCR was conducted on a real-time PCR system using SYBR Premix Ex Taq™ (Takara). Gene expression was analyzed using the following primer pairs: *Gli1* (forward: GAGGTTGGGATGAAGAAGCA, reverse: GTGCCAATC-CGGTGGAGTCAGACCC), *Ptch1* (forward: GTGGACGAGTGAGTCGAGAAT, reverse: GGAGTGCTGAGTCCAGGTGT), *Sostdc1* (forward: AGGCAGGCATTTCAGTAGCA, reverse: CTGGCCGTCCGAAATGTA), and *Hhip* (forward: GGCTCTGTCGAAACGGCTAC, reverse: GGCACTTGTTCGGTCTGACA). *Gapdh* was used as the internal control, with primer sequences as follows: forward: AACAGCAACTCCCACTCTTC, reverse: CCTGTTGCTGTAGCCGTATT. Relative gene expression was determined using the 2^−ΔΔCt^ method with QuantStudio Design and Analysis software.

## Supplementary information

Supplementary Information

## Data Availability

The raw and processed bulk RNA-Seq data associated with this study have been deposited in the NCBI GEO database under the accession number GSE290878.
