# Supplementary material for: Progressive tooth pattern changes in Cilk1-deficient mice depending on Hedgehog signaling
Source: Int J Oral Sci. 2025 Dec 1;17:71. doi: 10.1038/s41368-025-00405-4 (PMC12665794; doi:10.1038/s41368-025-00405-4)
Supplement: Supplementary file 1 — Supplementary Information [file 41368_2025_405_MOESM1_ESM.pdf]

Progressive Tooth Pattern Changes in *Cilk1*-Deficient mice Depending on Hedgehog Signaling

Minjae Kyeong[1*], Ju-Kyung Jeong[2*], Dinuka Adasooriya[1], Shiqi Kan[1], Jiwoo Kim[1], Jieun Song[3], Sihyeon Park[3], Su-Yeon Je[3], Seok Jun Moon[1,4], Young-Bum Park[5], Hyuk Wan Ko[3†], Eui-Sic Cho[2†], and Sung-Won Cho[1†]

[1]*Department of Oral Biology, BK21 FOUR Project, Oral Science Research Center, Yonsei University College of Dentistry, Seoul, Korea*

[2]*Cluster for Craniofacial Development and Regeneration Research, Institute of Oral Biosciences, Jeonbuk National University School of Dentistry, Jeonju, Korea*

[3]*Department of Biochemistry, Yonsei University College of Life Science and Biotechnology, Seoul, Korea*

[4]*Taste Research Center, Yonsei University College of Dentistry, Seoul, Korea*

[5]*Department of Prosthodontics, Yonsei University College of Dentistry, Seoul, Korea*

Supplementary Information

Including

- Supplementary Figure legends
- Supplementary Tables

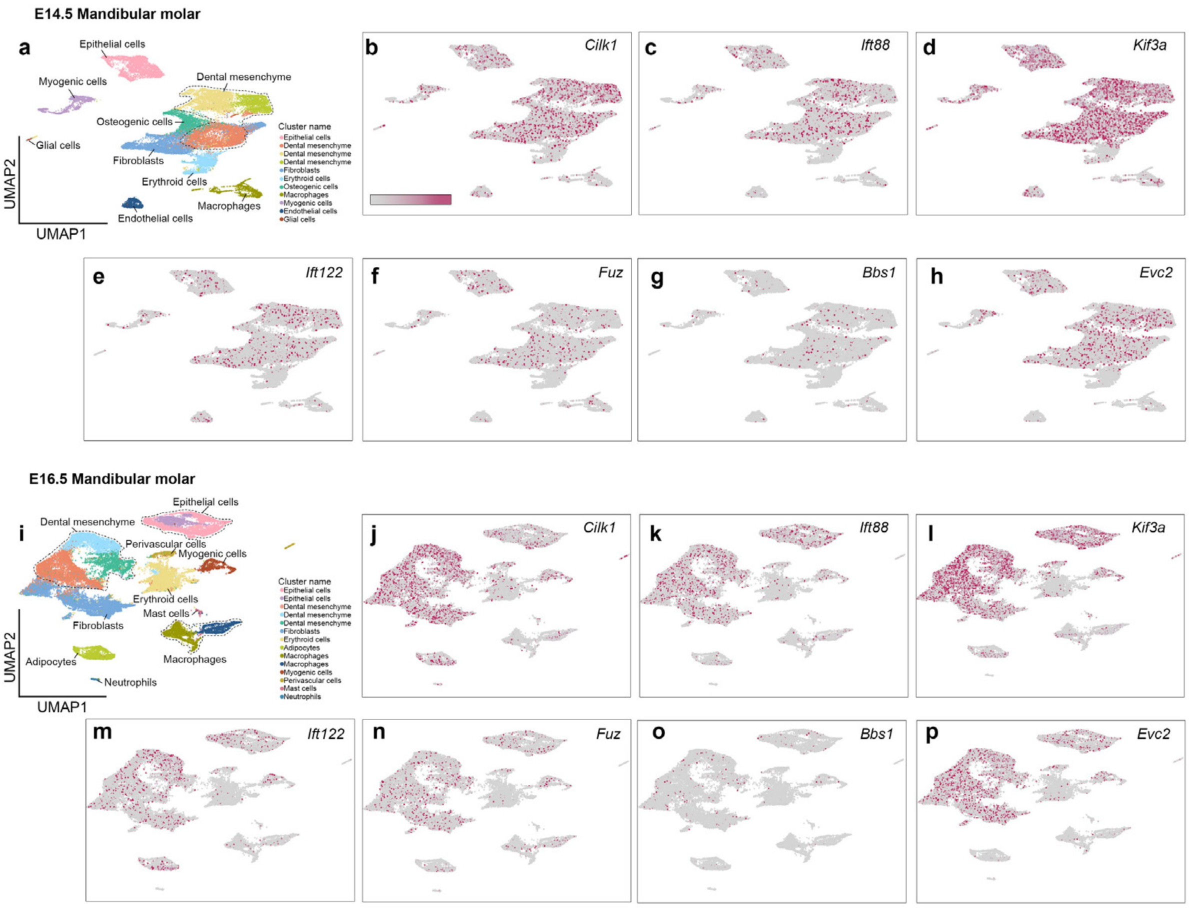

Supplementary Figure 1. Cell Clusters Expressing Cilia-Related Genes Detected from Single-Cell RNA Sequencing Analysis of Developing Molars

a–p Cell type clusters of mandibular molars at E14.5 (a) and E16.5 (i) displayed in UMAP plots. Cells that express the corresponding genes are indicated by highlighted red dots. *Cilk1* is expressed in many cell clusters throughout the tooth germ, but *Cilk1*-expressing cells are particularly abundant in the dental mesenchyme, which is similar to the expression pattern of other cilia-related genes, such as *Cilk1*, *Ift88*, *Kif3a*, *Ift122*, *Fuz*, *Bbs1*, and *Evc2*, at both E14.5 (b–h) and E16.5 (j–p).

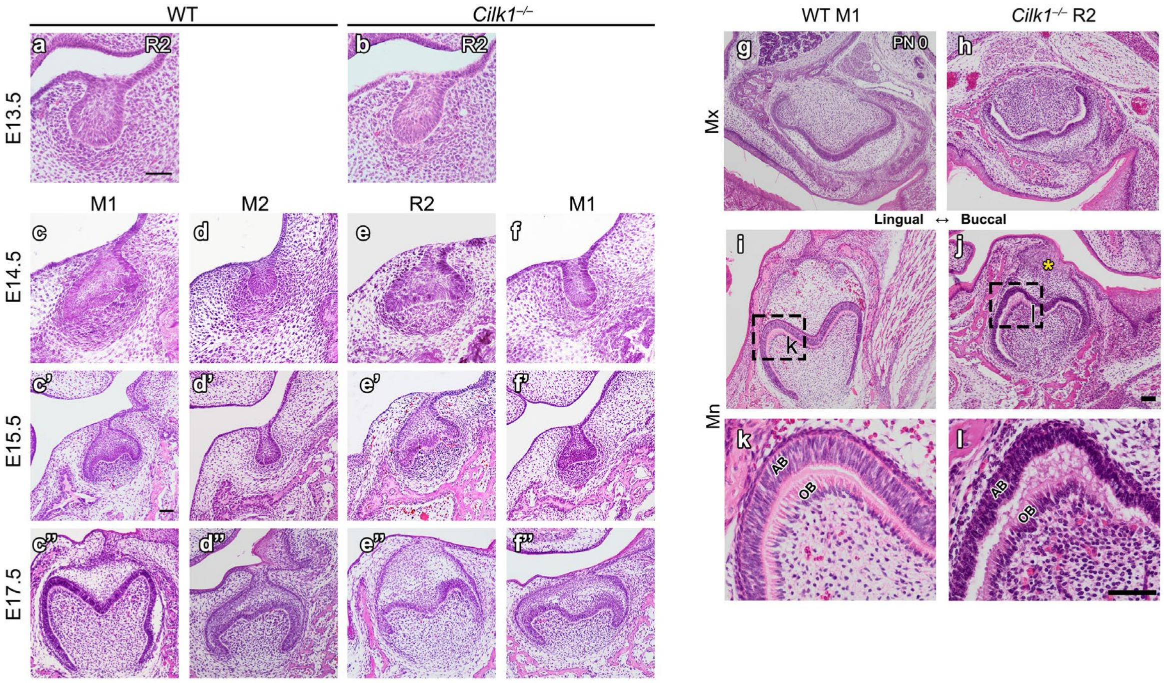

Supplementary Figure 2. Morphological Alteration in Tooth Germs of *Cilk1*$^{-/-}$ Mice

a–f'' Frontal histologic sections of diastemal supernumerary tooth (R2), first molar (M1) and second molar (M2) in wild-type (WT) and *Cilk1*$^{-/-}$ mice during development. At E13.5, the R2 is at the bud stage in both wild-type and *Cilk1*$^{-/-}$ mice (a, b). In wild-type mice, M1—into which R2 has been incorporated—progresses to the cap stage at E14.5 (c), the early bell stage at E15.5 (c'), and the late bell stage at E17.5 (c''). M2 in wild-type mice develops from the bud stage at E14.5 (d) to the cap stage at E15.5 (d'), and reaches the bell stage by E17.5 (d''). In *Cilk1*$^{-/-}$ mice, R2 survives and progresses to the cap stage at E14.5 (e), the early bell stage at E15.5 (e') and the bell stage at E17.5 (e''). M1 in *Cilk1*$^{-/-}$ mice is at the bud stage at E14.5 (f), advances to the cap stage at E15.5 (f'), and reaches the bell stage at E17.5 (f''). **g–j** At postnatal day 0 (PN 0), both mice show bell-stage appearance, but the dental epithelium of R2 in *Cilk1*$^{-/-}$ mice appears underdeveloped, particularly showing a thick dental lamina (yellow asterisk). **k–l** Ameloblasts (AB) and odontoblasts (OB) are observed as columnar cells in both mice but are shorter in the R2 of *Cilk1*$^{-/-}$ mice (l) than in the first molar of wild-type mice (k). Scale bars: 50 μm.

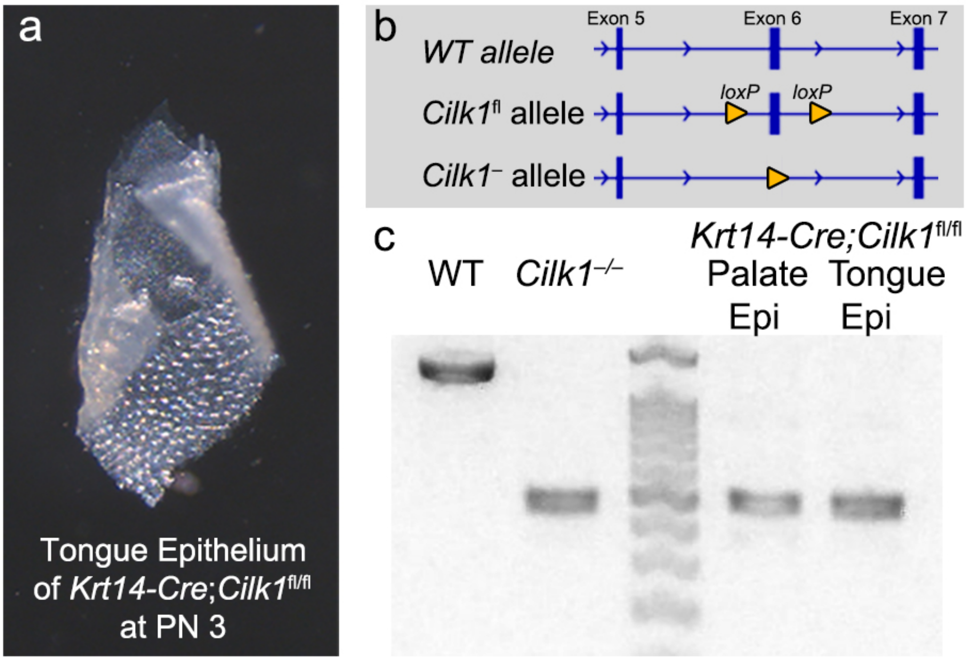

Supplementary Figure 3. Deletion of *Cilk1* Exon 6 in the Oral Epithelium of *Krt14-Cre;Cilk1*$^{fl/fl}$ Mice

a Tongue epithelium isolated from *Krt14-Cre;Cilk1*$^{fl/fl}$ mice at PN 3. **b** Schemes of wild-type (WT), floxed (*Cilk1*^{fl}), and knockout (*Cilk1*$^{–}$) alleles. LoxP sites flank exon 6 of the *Cilk1* in the floxed allele, which is excised upon Cre recombination to generate the knockout allele. **c** PCR analysis confirming deletion of exon 6 in whole tissue from *Cilk1*$^{–/–}$ mice and in epithelial tissue from the palate and tongue of *Krt14-Cre;Cilk1*$^{fl/fl}$ mice.

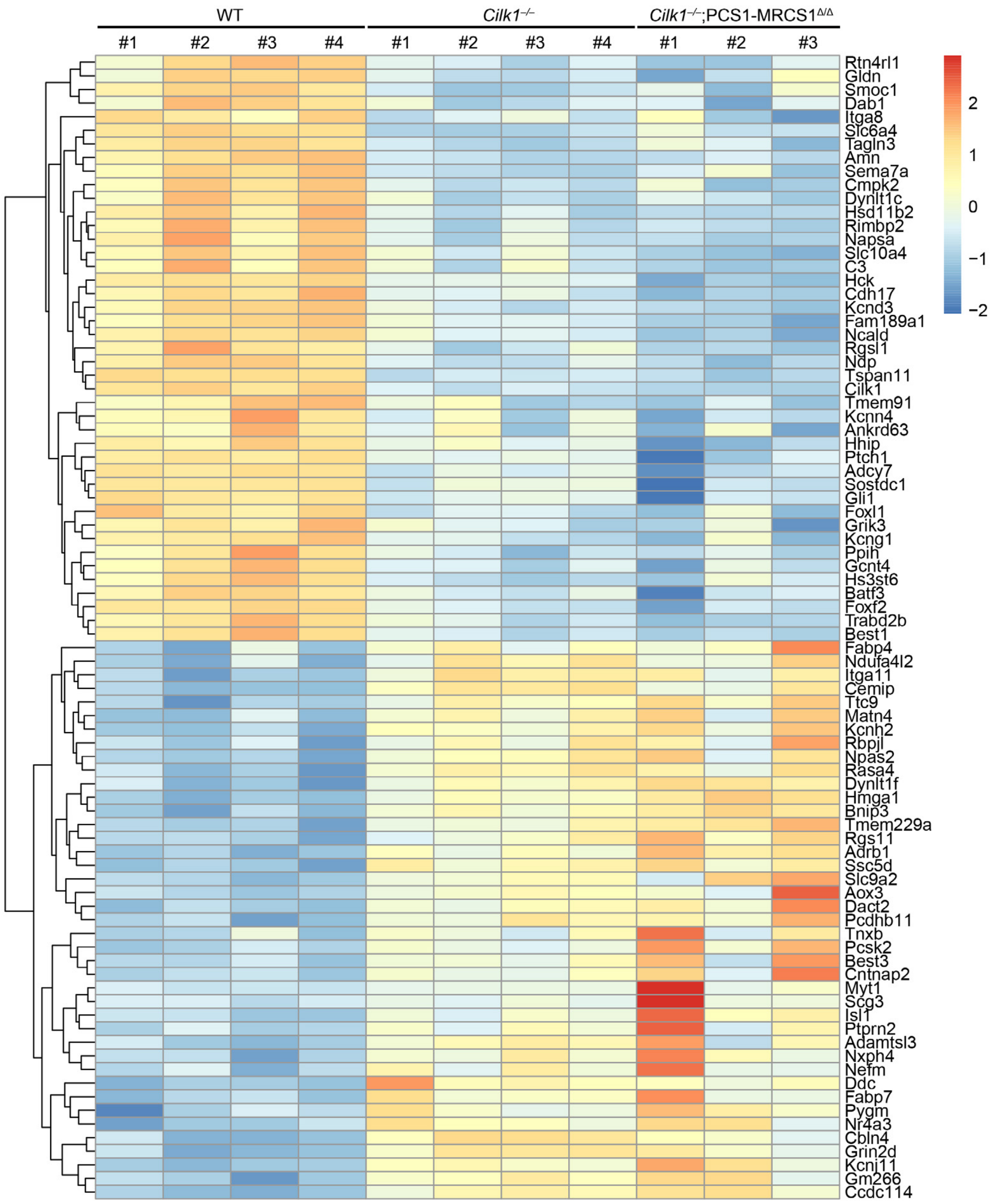

Supplementary Figure 4. Heatmap Showing Hierarchical Clustering of Differentially Expressed Genes

Normalized expression values of 43 commonly downregulated and 41 commonly upregulated genes in *Cilk1*$^{-/-}$ and *Cilk1*$^{-/-}$;PCS1−MRCS1$^{\triangle/\triangle}$ mice compared to wild-type controls are shown with red implying increased expression and blue showing decreased expression.

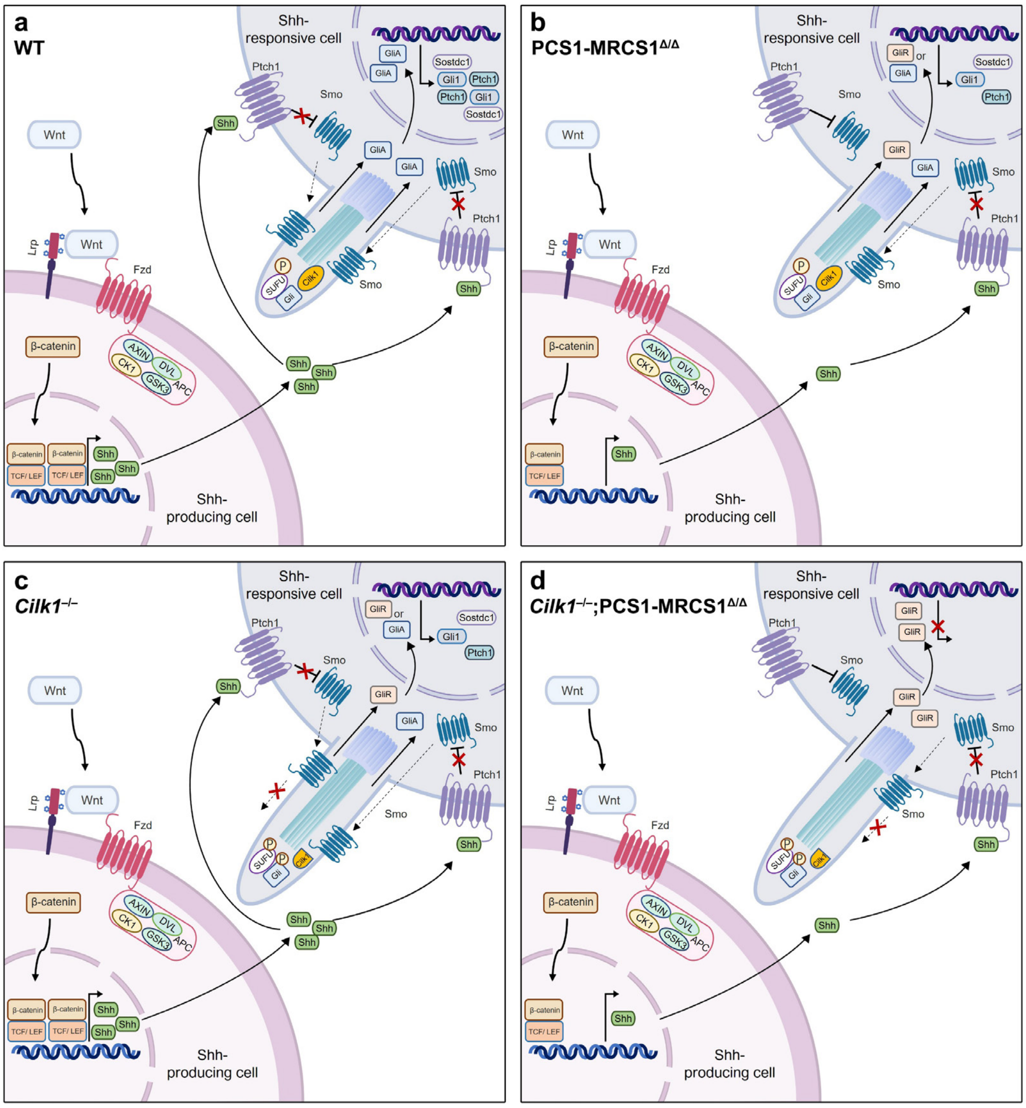

Supplementary Figure 5. Hedgehog signaling between Shh-producing and -responsive cells

a In wild-type (WT) mice, Sonic hedgehog (Shh) protein production and secretion are induced by Wnt signaling in Shh-producing cells. Shh proteins bind to the receptor Ptch1 on the membrane of Shh-responsive cells, translocating Smoothened (Smo) and processing of Gli proteins into activator (GliA) in primary cilia, of which the function is regulated by Cilk1. GliA then enters the nucleus to transcribe Hedgehog (Hh) signaling target genes. **b** In PCS1−MRCS1$^{\triangle/\triangle}$ mice, Shh-producing cells exhibit reduced Shh protein expression. **c** In *Cilk1*$^{-/-}$ mice, Shh-responsive cells fail to translocate Smoothened to the ciliary tip, even in the presence of Shh. **d** Synergistic inhibitory effects of Shh-production and primary cilia function on the Hh signaling activity in *Cilk1*$^{-/-}$;PCS1−MRCS1$^{\triangle/\triangle}$ mice.

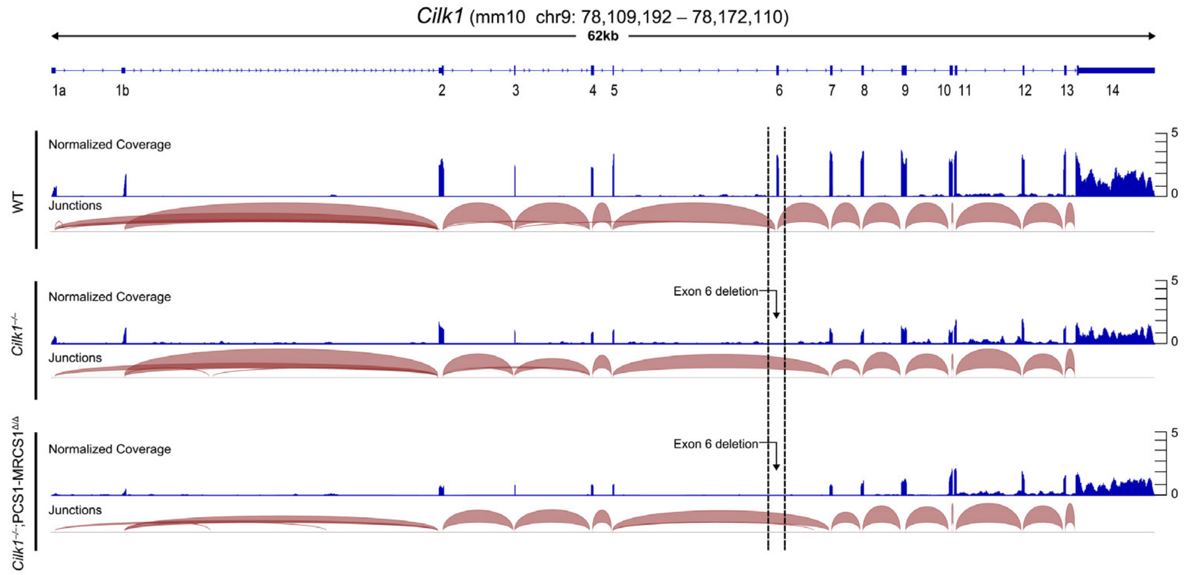

Supplementary Figure 6. RNA-seq read alignment profiles of *Cilk1* gene locus in WT, *Cilk1*$^{-/-}$ and *Cilk1*$^{-/-}$;PCS1−MRCS1$^{\triangle/\triangle}$ mice showing the deletion region

Blue peaks represent read depth across *Cilk1* gene exons, with the exon 6 region—deleted in *Cilk1*$^{-/-}$ and *Cilk1*$^{-/-}$;PCS1−MRCS1$^{\triangle/\triangle}$ mice—highlighted by dotted lines. Splice junction plots show normal arcs spanning exon 6 in WT, whereas these arcs are absent in *Cilk1*$^{-/-}$ and *Cilk1*$^{-/-}$;PCS1−MRCS1$^{\triangle/\triangle}$ mice, indicating altered splicing resulting from the deletion.

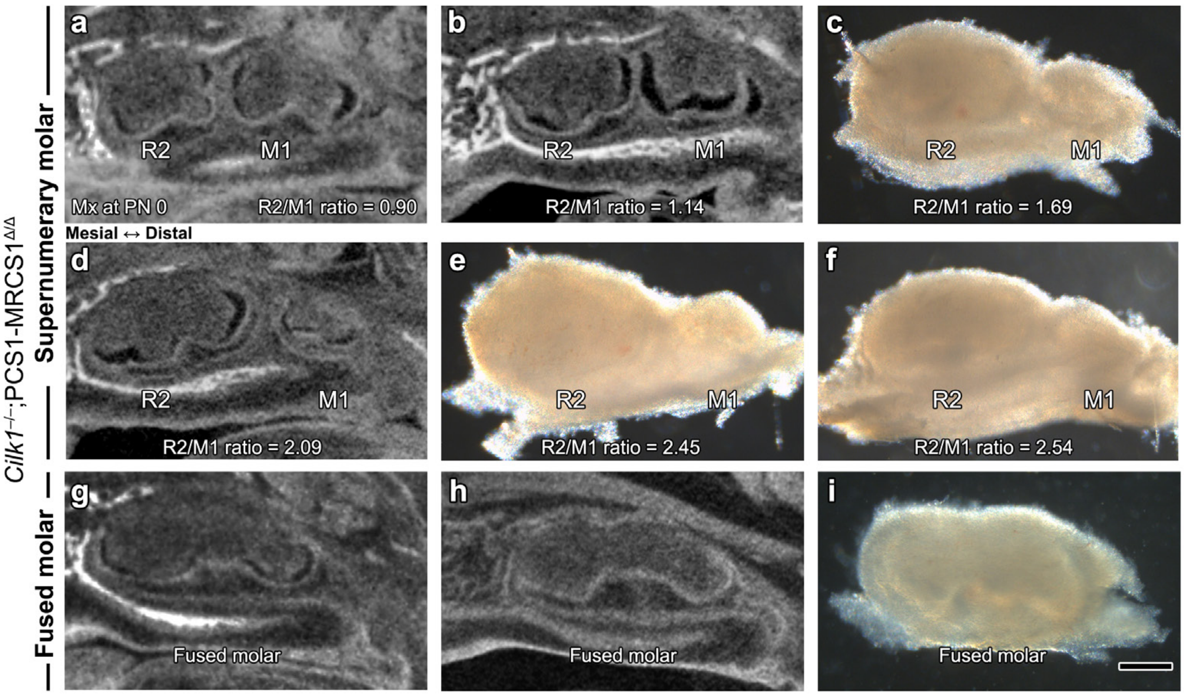

Supplementary Figure 7. Altered maxillary molar length ratio in *Cilk1*$^{-/-}$;PCS1−MRCS1$^{\triangle/\triangle}$ mice

Cilk1$^{-/-}$;PCS1−MRCS1$^{\triangle/\triangle}$ mice, an aberrant Shh-producing and -responsive model, exhibits altered molar patterning due to the influence on the relative growth of the diastemal supernumerary tooth (R2), leading to an increased R2/M1 ratio. **a, b, d** Micro-CT images of maxillary molars at postnatal day 0 (PN 0), illustrating differences in the relative size of R2 and first molar (M1). The Maxillary R2/M1 ratio is indicated above each panel. **c, e, f** Bright-field images of dissected molars from the same *Cilk1*$^{-/-}$;PCS1−MRCS1$^{\triangle/\triangle}$ mice, displaying differences in tooth proportions along the mesial-distal axes. **g-i** Fused molar phenotype observed in the maxilla of *Cilk1*$^{-/-}$;PCS1−MRCS1$^{\triangle/\triangle}$ mice Scale bar: a−f: 250 μm.

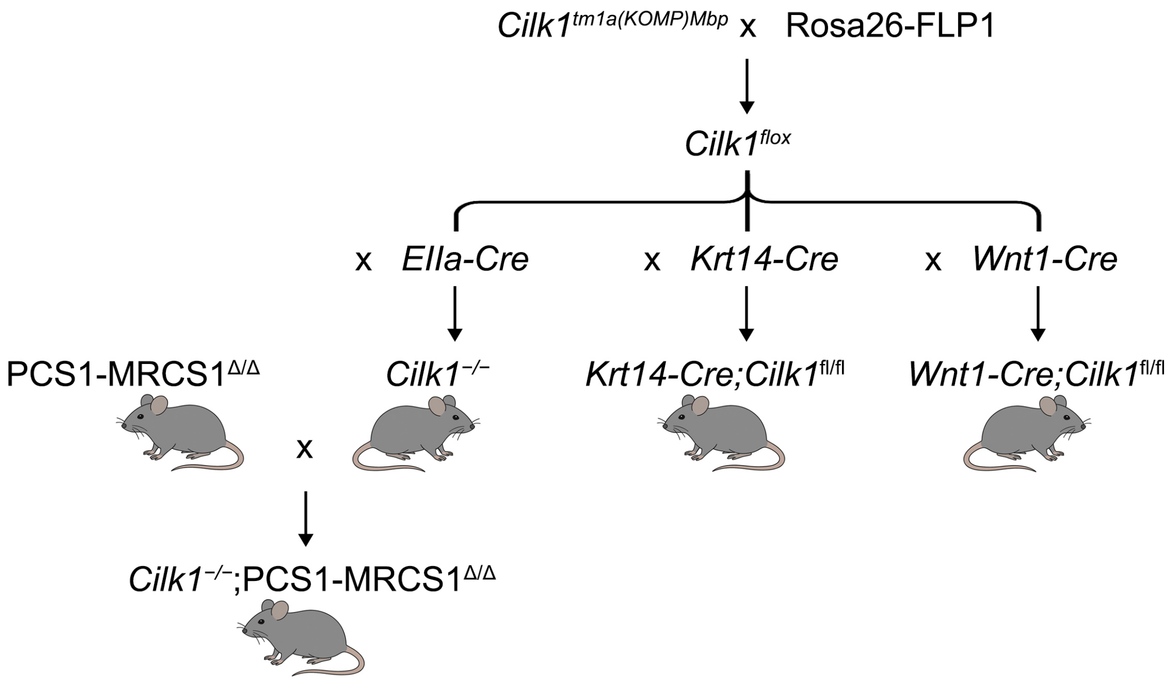

Supplementary Figure 8. Generation of *Cilk1* Knockout Mouse Models

Systemic and tissue-specific *Cilk1* knockout mice were generated using *EIIa-Cre, Krt14-Cre*, and *Wnt1-Cre* lines crossed with *Cilk1*$^{fl/fl}$ mice. *Cilk1*$^{-/-}$ mice were subsequently crossed with PCS1−MRCS1$^{\triangle/\triangle}$ mice to generate *Cilk1*$^{-/-}$;PCS1−MRCS1$^{\triangle/\triangle}$ mice. Mutant mice analyzed in this study are marked with mouse symbol.

Supplementary Table 1. Commonly upregulated genes in both *Cilk1*$^{-/-}$ and *Cilk1*$^{-/-}$;PCS1−MRCS1$^{\Delta/\Delta}$ mice compared to wild-type mice in maxillary tooth germs at E14.5 (fold change > 1.5, p value < 0.05).

Gene id	Gene name	Gene description	*Cilk1*$^{-/-}$ vs **WT**		*Cilk1*$^{-/-}$;PCS1−MRCS1$^{\Delta/\Delta}$ vs **WT**		*Cilk1*$^{-/-}$;PCS1−MRCS1$^{\triangle/\triangle}$ **vs** *Cilk1*$^{-/-}$	
			**Fold change**	**P value**	**Fold change**	**P value**	**Fold change**	**P value**
ENSMUSG00000070469	*Adamtsl3*	ADAMTS-like 3	2.162	0.013	2.732	0.019	1.264	0.585
ENSMUSG00000035283	*Adrb1*	adrenergic receptor, beta 1	2.382	0.001	4.535	0.000	1.904	0.065
ENSMUSG00000064294	*Aox3*	aldehyde oxidase 3	2.239	0.035	3.716	0.013	1.659	0.337
ENSMUSG00000020169	*Best3*	bestrophin 3	3.659	0.032	6.054	0.029	1.655	0.538
ENSMUSG00000078566	*Bnip3*	BCL2/adenovirus E1B interacting protein 3	1.511	0.000	1.826	0.000	1.208	0.153
ENSMUSG00000067578	*Cbln4*	Cerebellin 4 precursor protein	9.596	0.000	5.260	0.001	0.548	0.230
ENSMUSG00000040189	*Ccdc114*	outer dynein arm docking complex subunit 1	1.559	0.001	1.698	0.004	1.089	0.639
ENSMUSG00000052353	*Cemip*	cell migration inducing protein, hyaluronan binding	2.625	0.000	1.947	0.011	0.742	0.253

ENSMUSG00000039419	*Cntnap2*	contactin associated protein-like 2	2.182	0.039	3.997	0.008	1.832	0.243
ENSMUSG00000048826	*Dact2*	dishevelled-binding antagonist of beta-catenin 2	1.536	0.011	1.937	0.005	1.261	0.319
ENSMUSG00000020182	*Ddc*	dopa decarboxylase	1.633	0.002	1.558	0.044	0.954	0.829
ENSMUSG00000095677	*Dynlt1f*	dynein light chain Tctex-type 1F	2.059	0.000	2.986	0.000	1.450	0.175
ENSMUSG00000062515	*Fabp4*	fatty acid binding protein 4, adipocyte	2.619	0.015	4.002	0.011	1.528	0.434
ENSMUSG00000019874	*Fabp7*	fatty acid binding protein 7, brain	2.267	0.020	3.419	0.012	1.508	0.399
ENSMUSG00000010529	*Gm266*	Predicted gene 266	1.557	0.008	1.753	0.015	1.125	0.610
ENSMUSG00000002771	*Grin2d*	glutamate receptor, ionotropic, NMDA2D (epsilon 4)	1.866	0.000	1.564	0.040	0.838	0.416
ENSMUSG00000046711	*Hmga1*	high mobility group AT-hook 1	1.556	0.000	2.117	0.000	1.361	0.012
ENSMUSG00000042258	*Isl1*	ISL1 transcription factor, LIM/homeodomain	3.029	0.015	14.100	0.000	4.656	0.015
ENSMUSG00000032243	*Itga11*	integrin alpha 11	2.008	0.000	1.829	0.013	0.910	0.700
ENSMUSG00000038319	*Kcnh2*	potassium voltage-gated channel, subfamily H (eag-related), member 2	1.965	0.002	2.430	0.003	1.237	0.473
ENSMUSG00000096146	*Kcnj11*	potassium inwardly rectifying channel, subfamily J, member 11	2.142	0.022	3.476	0.007	1.623	0.291
ENSMUSG00000016995	*Matn4*	matrilin 4	2.345	0.021	3.243	0.021	1.383	0.525
ENSMUSG00000010505	*Myt1*	myelin transcription factor 1	6.257	0.033	72.092	0.000	11.522	0.037
ENSMUSG00000040280	*Ndufa4l2*	Ndufa4, mitochondrial complex associated like 2	2.013	0.001	1.867	0.032	0.928	0.797
ENSMUSG00000022054	*Nefm*	neurofilament, medium polypeptide	6.375	0.039	31.621	0.006	4.960	0.197
ENSMUSG00000026077	*Npas2*	neuronal PAS domain protein 2	1.978	0.003	2.111	0.019	1.067	0.838
ENSMUSG00000028341	*Nr4a3*	nuclear receptor subfamily 4, group A, member 3	2.314	0.013	2.582	0.041	1.116	0.813
ENSMUSG00000040258	*Nxph4*	neurexophilin 4	2.808	0.012	4.598	0.007	1.638	0.382
ENSMUSG00000051486	*Pcdhb11*	protocadherin beta 11	1.634	0.015	1.806	0.033	1.105	0.717
ENSMUSG00000027419	*Pcsk2*	proprotein convertase subtilisin/kexin type 2	3.172	0.005	7.425	0.000	2.341	0.123
ENSMUSG00000056553	*Ptprn2*	protein tyrosine phosphatase receptor type N polypeptide 2	3.947	0.046	8.979	0.021	2.275	0.383
ENSMUSG00000032648	*Pygm*	muscle glycogen phosphorylase	1.626	0.050	1.977	0.046	1.216	0.567
ENSMUSG00000004952	*Rasa4*	RAS p21 protein activator 4	1.511	0.002	1.508	0.027	0.998	0.992
ENSMUSG00000017007	*Rbpjl*	recombination signal binding protein for immunoglobulin kappa J region-like	2.536	0.026	3.164	0.044	1.248	0.697
ENSMUSG00000024186	*Rgs11*	regulator of G-protein signaling 11	1.521	0.012	1.939	0.004	1.275	0.289
ENSMUSG00000032181	*Scg3*	secretogranin III	5.608	0.033	32.517	0.001	5.798	0.105
ENSMUSG00000026062	*Slc9a2*	solute carrier family 9 (sodium/hydrogen exchanger), member 2	1.864	0.023	2.216	0.034	1.189	0.644
ENSMUSG00000035279	*Ssc5d*	scavenger receptor cysteine rich family, 5 domains	1.788	0.001	2.065	0.002	1.155	0.538
ENSMUSG00000048022	*Tmem229a*	transmembrane protein 229A	1.636	0.005	2.233	0.001	1.365	0.193
ENSMUSG00000033327	*Tnxb*	tenascin XB	4.507	0.024	6.635	0.037	1.472	0.666
ENSMUSG00000042734	*Ttc9*	tetratricopeptide repeat domain 9	1.529	0.015	1.730	0.021	1.132	0.602

Supplementary Table 2. Commonly downregulated genes in both *Cilk1*$^{-/-}$ and *Cilk1*$^{-/-}$;PCS1−MRCS1$^{\Delta/\Delta}$ mice compared to wild-type mice in maxillary tooth germs at E14.5 (fold change < -1.5, p value < 0.05).

**Gene id**	**Gene name**	**Gene description**	*Cilk1*$^{-/-}$ vs **WT**		*Cilk1*$^{-/-}$;PCS1−MRCS1$^{\Delta/\Delta}$ vs **WT**		*Cilk1*$^{-/-}$;PCS1−MRCS1$^{\Delta/\Delta}$ **vs** *Cilk1*$^{-/-}$	
			**Fold change**	**P value**	**Fold change**	**P value**	**Fold change**	**P value**
ENSMUSG00000031659	*Adcy7*	adenylate cyclase 7	-1.759	0.000	-2.283	0.000	-1.298	0.178
ENSMUSG00000021278	*Amn*	Amnionless	-3.698	0.000	-2.905	0.006	-0.786	0.546
ENSMUSG00000078137	*Ankrd63*	Ankyrin repeat domain 63	-2.161	0.022	-2.457	0.050	-1.137	0.779
ENSMUSG00000026630	*Batf3*	Basic leucine zipper transcription factor, ATF-like 3	-1.734	0.020	-2.272	0.012	-1.311	0.409
ENSMUSG00000037418	*Best1*	Bestrophin 1	-2.277	0.000	-2.785	0.000	-1.223	0.436
ENSMUSG00000024164	*C3*	Complement component 3	-3.185	0.002	-7.309	0.000	-2.295	0.102
ENSMUSG00000028217	*Cdh17*	Cadherin 17	-2.558	0.000	-3.524	0.000	-1.377	0.301
ENSMUSG00000009828	*Cilk1*	ciliogenesis associated kinase 1	-1.771	0.000	-1.999	0.000	-1.129	0.197
ENSMUSG00000020638	*Cmpk2*	Cytidine/uridine monophosphate kinase 2	-1.654	0.004	-1.709	0.025	-1.033	0.892
ENSMUSG00000028519	*Dab1*	Disabled 1	-1.753	0.008	-1.980	0.020	-1.130	0.677
ENSMUSG00000000579	*Dynlt1c*	Dynein light chain Tctex-type 1C	-1.534	0.002	-1.575	0.020	-1.027	0.893
ENSMUST00000253234	*Fam189a1*	Novel transcript, antisense to Fam189a1.	-1.549	0.004	-2.259	0.000	-1.459	0.072
ENSMUSG00000038402	*Foxf2*	Forkhead box F2	-2.658	0.000	-3.202	0.000	-1.205	0.466
ENSMUSG00000097084	*Foxl1*	Forkhead box L1	-2.986	0.006	-3.023	0.044	-1.012	0.982
ENSMUSG00000091387	*Gcnt4*	glucosaminyl (N-acetyl) transferase 4, core 2 (beta-1,6-N-acetylglucosaminyltransferase)	-2.309	0.000	-2.455	0.003	-1.063	0.839
ENSMUSG00000046167	*Gldn*	gliomedin	-2.666	0.002	-2.337	0.028	-0.877	0.734
ENSMUSG00000025407	*Gli1*	GLI-Kruppel family member GLI1	-1.912	0.000	-2.507	0.000	-1.311	0.274
ENSMUSG00000001985	*Grik3*	Glutamate receptor, ionotropic, kainate 3	-2.638	0.011	-3.134	0.030	-1.188	0.744
ENSMUSG00000003283	*Hck*	Hemopoietic cell kinase	-1.859	0.000	-3.004	0.000	-1.616	0.007
ENSMUSG00000064325	*Hhip*	Hedgehog-interacting protein	-1.515	0.003	-2.360	0.000	-1.558	0.021
ENSMUSG00000039628	*Hs3st6*	Heparan sulfate (glucosamine) 3-O-sulfotransferase 6	-2.184	0.000	-1.921	0.008	-0.880	0.606
ENSMUSG00000031891	*Hsd11b2*	Hydroxysteroid 11-beta dehydrogenase 2	-2.246	0.000	-2.419	0.004	-1.077	0.812
ENSMUSG00000026768	*Itga8*	Integrin alpha 8	-1.556	0.010	-1.610	0.046	-1.035	0.886
ENSMUSG00000040896	*Kcnd3*	potassium voltage-gated channel, Shal-related family, member 3	-1.610	0.002	-1.942	0.002	-1.206	0.376
ENSMUSG00000074575	*Kcng1*	potassium voltage-gated channel, subfamily G, member 1	-2.143	0.005	-2.230	0.031	-1.041	0.915
ENSMUSG00000054342	*Kcnn4*	potassium intermediate/small conductance calcium-activated channel, subfamily N, member 4	-1.851	0.003	-2.192	0.005	-1.184	0.550
ENSMUSG00000002204	*Napsa*	Napsin A aspartic peptidase	-3.058	0.000	-4.161	0.001	-1.361	0.461
ENSMUSG00000051359	*Ncald*	Neurocalcin delta	-1.627	0.000	-2.330	0.000	-1.432	0.052
ENSMUSG00000040138	*Ndp*	Norrie disease (pseudoglioma) (human)	-2.372	0.000	-3.030	0.000	-1.277	0.310
ENSMUSG00000060288	*Ppih*	Peptidyl prolyl isomerase H	-1.759	0.002	-1.790	0.022	-1.017	0.946
ENSMUSG00000021466	*Ptch1*	Patched 1	-1.909	0.001	-2.968	0.000	-1.554	0.101
ENSMUSG00000042641	*Rgsl1*	Regulator of G-protein signaling like 1	-2.049	0.019	-2.509	0.027	-1.225	0.628
ENSMUSG00000029420	*Rimbp2*	RIMS binding protein 2	-2.763	0.000	-2.906	0.007	-1.051	0.899

ENSMUSG00000045287	*Rtn4rl1*	Reticulon 4 receptor-like 1	-1.834	0.002	-2.251	0.003	-1.227	0.456
ENSMUSG00000038264	*Sema7a*	sema domain, immunoglobulin domain (Ig), and GPI membrane anchor, (semaphorin) 7A	-1.980	0.000	-1.784	0.006	-0.901	0.620
ENSMUSG00000029219	*Slc10a4*	solute carrier family 10 (sodium/bile acid cotransporter family), member 4	-2.130	0.004	-4.647	0.000	-2.181	0.033
ENSMUSG00000020838	*Slc6a4*	solute carrier family 6 (neurotransmitter transporter, serotonin), member 4	-2.267	0.000	-1.859	0.033	-0.820	0.499
ENSMUSG00000021136	*Smoc1*	SPARC related modular calcium binding 1	-2.066	0.000	-1.706	0.028	-0.826	0.430
ENSMUSG00000036169	*Sostdc1*	Sclerostin domain containing 1	-1.708	0.001	-2.395	0.000	-1.403	0.146
ENSMUSG00000022658	*Tagln3*	Transgelin 3	-1.721	0.000	-1.573	0.024	-0.910	0.638
ENSMUSG00000061702	*Tmem91*	Transmembrane protein 91	-2.478	0.010	-2.859	0.029	-1.154	0.767
ENSMUSG00000070867	*Trabd2b*	TraB domain containing 2B	-1.654	0.000	-2.010	0.000	-1.215	0.305
ENSMUSG00000030351	*Tspan11*	Tetraspanin 11	-1.711	0.000	-1.864	0.000	-1.090	0.395
